# Particle size-related limitations of persistent phosphors based on the doped Y_3_Al_2_Ga_3_O_12_ system

**DOI:** 10.1038/s41598-020-80335-9

**Published:** 2021-01-08

**Authors:** Vitalii Boiko, Zhengfa Dai, Marta Markowska, Cristina Leonelli, Cecilia Mortalò, Francesco Armetta, Federica Ursi, Giorgio Nasillo, Maria Luisa Saladino, Dariusz Hreniak

**Affiliations:** 1 Institute of Low Temperature and Structure Research, Polish Academy of Sciences, Okólna 2, 50-422 Wrocław, Poland; 2 Department of Engineering “Enzo Ferrari”, University of Modena and Reggio Emilia, Via Pietro Vivarelli 10, 41125 Modena, Italy; 3 Istituto di Chimica della Materia Condensata e di Tecnologie per l’Energia-ICMATE, Consiglio Nazionale delle Ricerche, Corso Stati Uniti 4, 35127 Padova, Italy; 4 Department of Biological, Chemical and Pharmaceutical Sciences and Technologies (STEBICEF) and INSTM UdR-Palermo, University of Palermo, Viale delle Scienze, Bld. 17, 90128 Palermo, Italy; 5 ATeN Center, University of Palermo, Viale delle Scienze, Bld. 18, 90128 Palermo, Italy

**Keywords:** Optical materials, Nanoparticle synthesis, Nanoparticles

## Abstract

Co-doped Ce^3+^, Cr^3+^ and Pr^3+^ yttrium–aluminium–gallium garnet powders of various sizes were obtained by co-precipitation method. The microstructure and morphology were investigated by XRPD, TEM and gas porosimetry. The luminescence properties were studied by excitation and emission spectra, quantum yield and decay times. Thermoluminescence measurements were performed to evaluate the activation energy, traps redistribution and frequency factor. Limitation in the energy transfer between dopant ions in the small particles, traps depth and surface defects were considered and investigated as responsible for the quenching of persistent luminescence. The phosphors annealed at 1100 °C show the optimal persistent luminescence and nano-particle size.

## Introduction

Persistent luminescence (PersL) nanophosphors activated by doping with lanthanide and transition metal ions have recently gained much attention due to their promising applications in the fields of bioimaging^1,2^, optical nano-thermometry^3^, solar-blind glowing tags in bright daylight^4^, safety displays^5,6^, etc. Many research groups around the world are strongly motivated to develop new hosts for such phosphors and new synthetic routes^5–9^. Appropriately doped *yttrium–aluminum-gallium* *garnet* (Y_3_Al_2_Ga_3_O_12_, YAGG) is known as one of the new host matrices for persistent phosphors, which can show continuous PersL lasting from minutes to even hours after ceasing the excitation source^8,10–12^. The most recent studies are devoted to the nanophosphors emitting in red and near-infrared (NIR) regions^2,13–16^. Phosphors with such properties may be obtained by co-doping materials with Cr^3+^ and other trivalent ions, but still the intensity and duration of PersL in the red region cannot compete with green and blue ones^14,15,17,18^. Polycrystalline YAGG co-doped with Cr^3+^ and Ce^3+^ (YAGG:Ce^3+^,Cr^3+^) has been intensively studied by the group of Tanabe^19–21^, and it was proposed for the acquisition of long PersL of Ce^3+^ at room temperature. The development of this system by adding a third dopant to obtain red and NIR PersL is of current interest^13–15,17,18^. In fact, the addition of some lanthanide ions has been already studied in order to obtain their PersL due to energy transfer (ET) from Ce^3+^ and Cr^3+^ ^11,22^. Special attention has been put to the investigation of the mechanism involved in the PersL, in particular the electron and hole traps creation^7,21,23–26^. Moreover, even if systematic studies have been carried out for microsized samples prepared by solid-state reactions, the size effect on the PersL is not well known in nano and sub-micron sized particles^1,5^. For this reason, the synthesis of enough high quality doped-YAGG nanophosphors is still a challenge and the current subject of research. Most of the investigated doped-YAGG nanophosphors are obtained by combustion and Pechini methods^14,27,28^. Only few authors used a so-called wet-chemical methods such as co-precipitation, hydro- solvo-thermal or sol–gel with the aim of controlling the phase purity, to tailor the particles size and agglomeration degree^15,28–30^.

The aim of this work is to correlate microstructure and morphology of YAGG:Ce^3+^,Cr^3+^,Pr^3+^ nanophosphors with their luminescence properties. YAGG nanophosphors were synthesized by co-precipitation and then annealed at various temperatures. The undoped YAGG and singly doped with Ce^3+^, Cr^3+^ or with Pr^3+^ were synthesized as references at the same experimental conditions in order to investigate the impact of co-doping and the effect of thermal treatment on the phase stability of crystallization products, their microstructure, particles size and agglomeration. The set of co-doping ions was chosen to verify their applicability as persistent phosphors and to obtain already proved efficient energy downshifting from ultraviolet (UV) and blue light to red and NIR regions^13–15,17,18^. In this system, Ce^3+^ ions should absorb UV and blue light and then transfer the absorbed energy to Cr^3+^ and Pr^3+^ ions leading to red and NIR emission. The efficient ET should be able to enhance the effectiveness of luminescent solar concentrators by exploiting the UV/blue conversion of solar energy to the maximum of the spectral absorption of the photovoltaic substrate.

## Experimental part

### Materials

Y(NO_3_)_3_·6H_2_O (Aldrich, 99.8%), Al(NO_3_)_3_·9H_2_O (Aldrich, 98%), Ga(NO_3_)_3_·9H_2_O (Aldrich, 99.9%), Pr_6_O_11_ (Sigma–Aldrich, 99.99%), Ce(NO_3_)_3_·6H_2_O (Aldrich, 99.99%), Cr(NO_3_)_3_·9H_2_O (Fluka, 97%), ammonia solution (Aldrich, 28%) and nitric acid (Aldrich, 90%) were used as starting materials. All solutions were prepared using chemicals as received and adding deionized water (conductivity 1.5 μS/m).

### Preparation of samples

Undoped (YAGG), singly doped (YAGG:Ce^3+^, YAGG:Cr^3+^, YAGG:Pr^3+^) and triply doped (YAGG:Ce^3+^,Cr^3+^,Pr^3+^) nanophosphors were prepared by the co-precipitation method following the procedure previously used for synthesis of YAG nanopowders^31–35^. First, nitrates were dissolved in deionized water and Pr_6_O_11_ was dissolved in dilute nitric acid in order to obtain praseodymium nitrate. For the synthesis of undoped Y_3_Al_2_Ga_3_O_12_ powders, yttrium, aluminum and gallium nitrates were mixed to obtain Y:Al:Ga atomic ratio equal to 3:2:3. The concentration of Al(NO_3_)_3_ was 0.5 mol L^−1^.

Nitrates were mixed to obtain the following atomic ratios: (Y + Ce):Al:Ga, (Y + Pr):Al:Ga, Y:(Al + Cr):Ga and (Y + Ce + Pr):(Al + Cr):Ga equal to 3:2:3 and the Y_2.994_Ce_0.006_Al_2_Ga_3_O_12_, Y_2.9925_Pr_0.0075_Al_2_Ga_3_O_12_, Y_3_Al_1.9875_Cr_0.0125_Ga_3_O_12_, and Y_2.9865_Ce_0.006_Pr_0.0075_Al_1.9875_Cr_0.0125_Ga_3_O_12_ formula. In the obtained samples, Ce^3+^ and Pr^3+^ ions substituted Y^3+^ ions with concentration 0.20 and 0.25 at.% in respect to Y^3+^ ions, and Cr^3+^ substituted Al^3+^ ions with concentration 0.63 at.% in respect to Al^3+^ ions.

The hydroxides were precipitated by dropwise addition of 0.5 mol L^−1^ ammonia solution until a pH equal to 8 was reached. The colloidal solution was thus maintained at pH 8 for 2 h. The white gelatinous precipitate was filtered and washed with deionized water to remove residual ammonia and nitrate ions. Finally, it was washed with ethanol and then was dried at 70 °C in the oven until it reached a constant weight. In order to obtain a crystalline powder, the dried powders were annealed at 900, 1000, 1100 and 1200 °C for 1 h in air with a synthesis yield of 80%. After the thermal treatment, undoped YAGG as well as YAGG:Cr^3+^, YAGG:Pr^3+^ powders were white, while the doped YAGG:Ce^3+^ , and YAGG:Ce^3+^,Cr^3+^, Pr^3+^ were green and light green tint, respectively.

#### Characterization techniques

*X-ray* *powder* *diffraction* *(XRPD)* *patterns* were acquired by a PANalytical X’Pert pro X-ray powder diffractometer at 40 kV and 30 mA in the 2*θ* range of 10°–80° (2*θ* step: 0.02626°) using nickel-filtered Cu K_α1_ radiation. The phase identification was performed using the X’pert HighScore Software. Phase composition, cell parameters, crystallite sizes and microstrain of the phosphors were evaluated by using the Rietveld method^36^ and the MAUD software^37^. N_2_ adsorption–desorption isotherms were registered at 77° K using a Quantachrome NOVA 2200 Multi-Station High Speed Gas Sorption Analyzer. Samples were outgassed at 200 °C for 2 h in the degas station. Adsorbed nitrogen volumes were normalized to the standard temperature and pressure. The specific surface area (S_BET_) was calculated according to the standard B.E.T. method in the relative absorption pressure (P/P_0_) range from 0.045 to 0.250^38^. The average particle size (D_BET_) of the phosphors was calculated based on the S_BET_^39^. Transmission electron microscope (TEM) micrographs of nanophosphors annealed at 900 and 1200 °C were acquired using a JEM-2100 (JEOL, Japan) Electron Microscope operating at a 200 kV accelerating voltage. Each powder was homogeneously dispersed in isopropanol by sonication for 2 min. A drop of the suspension was deposited on a lacey-carbon grid of 300 meshes. After complete solvent evaporation, the grid was introduced into the TEM chamber for analysis. TEM micrographs of nanophosphors annealed at 1000 and 1100 °C were acquired using a high resolution scanning/transmission electron (S/TEM) microscope (Thermo Scientific Talos F200S) equipped with energy dispersive X-ray spectroscopy (EDS) and operating at 200 kV accelerating voltage. Samples were homogeneously dispersed in bi-distilled water (Millipore) by sonication for two minutes. A drop of each suspension was deposited on a copper grid of 200 mesh coated with a transparent polymer (Formvar/carbon) and then dried. Subsequently, specimens were carbonated (Carbon Coater—Balzers CED-010) for TEM investigations. Photoluminescence emission (PL) and excitation (PLE) spectra were measured using the FLS980 Fluorescence Spectrometer from Edinburgh Instruments equipped with 450 W Xenon lamp as an excitation source. Excitation arm was supplied with holographic grating of 1800 lines/mm, blazed at 300 nm, while the emission arm was supplied with ruled grating, 1800 lines/mm blazed at 500 nm. The R928P side window photomultiplier tube from Hamamatsu was used as a detector. The scanning range was from 250 to 680 nm for the PLE spectrum and 460 to 820 nm for PL spectrum with spectral resolution of 0.1 nm. The lifetime measurements were carried out using a femtosecond laser setup composed of a Coherent Libra-S all-in-one ultrafast oscillator and regenerative amplifier laser system, with pulse duration less than 100 fs at 1 kHz repetition rate, a Coherent OPerA-Solo optical parametric amplifier and a Hamamatsu C5677 streak camera with time resolution of 14 ps. Quantum efficiency of luminescence was measured using the same equipment as PL and PLE equipped with integrating sphere with diameter 30 cm. Measurement was performed for the pellets with a diameter of 5 mm and mass 1.5 mg prepared using press (pressure: 15 kN) from all synthesized powders. The PersL spectra of the YAGG nanophosphors were measured at room temperature after 450 nm LED irradiation for 5 min using Si CCD spectrometer (USB2000+, Ocean Optics), integration time 1 s. The detected spectra intensity was calibrated to a standard halogen lamp. The *PersL* *decay* *curves* and thermoluminescence (*TL*) *curves* were measured with a lexsyg research—fully automated TL/OSL Reader (Freiberg Instruments). The signal was collected with a R13456 PMT (Hamamatsu Photonics) monitoring the global emission from the whole spectral response (from 185 to 980 nm) with an integration (channel) time of 0.1 s. The TL glow curves were collected from room temperature to 300 °C with heating rate 2, 1, 0.75, 0.5 and 0.25 °C s^−1^. Blue Laser Diode PL 450B (λ_max_ = 450 nm, FWHM = 2 nm) by Osram and Varian VF-50J/S RTG tube with tungsten core and copper case as an X-ray radiation source were used as irradiation sources. The excitation intensity of the LD was 1 mW/cm^2^ at sample position. The voltage and amperage for the X-Ray source were 15 kV and 0.1 mA, respectively. The persistent phosphors and ethanol were homogeneously mixed by using ultrasonics to obtain slurry. The homogeneous slurry was dripped on a metal holder and then the metal holder was heated to 100 °C and kept for 10 min to get rid of the solvent and form a thin film. For each phosphor annealed at different temperatures, approximately the same mass of sample was placed on the metal holder to obtain the film with almost the same thickness and realize the same PersL measurement condition. The samples thus prepared were placed in a TL instrument and the TL glow curves and PersL decay curves were collected. Finally, the TL and PersL intensities were normalized according to the mass of the sample.

## Results and discussion

### Microstructural and morphological investigation

**Figure 1:**
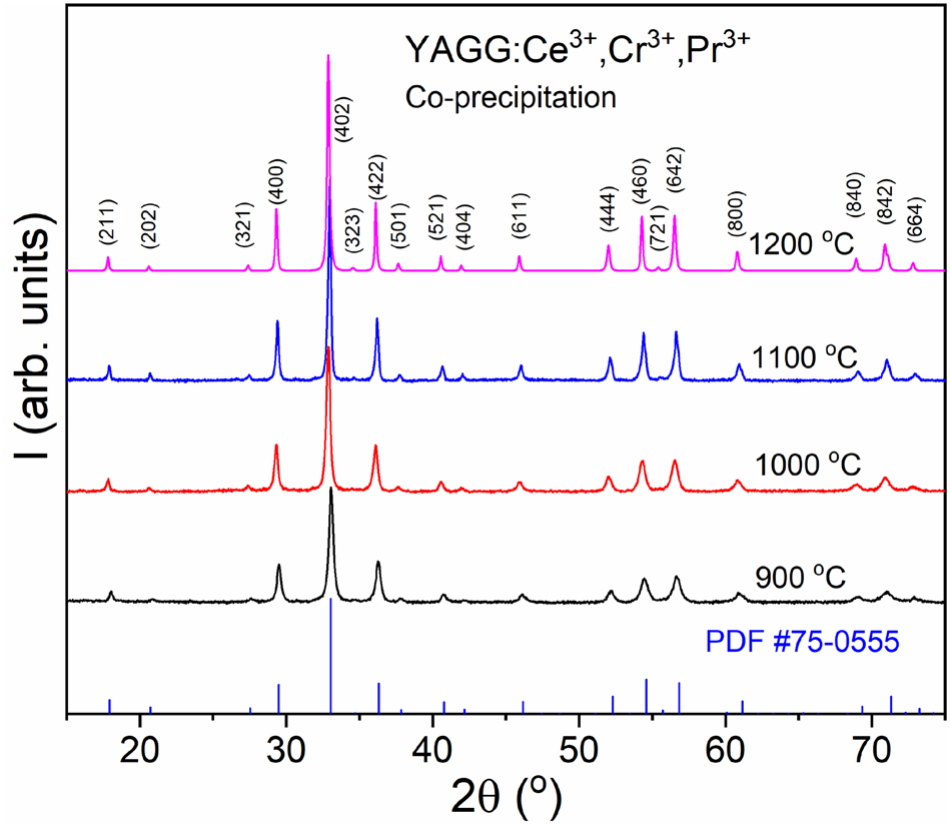
XRPD patterns of YAGG:Ce^3+^,Cr^3+^,Pr^3+^ annealed at different temperatures in air.

The XRPD patterns of YAGG nanophosphors co-doped with Ce^3+^, Cr^3+^, Pr^3+^ are reported in Fig. 1. The XRPD patterns of undoped YAGG as well as YAGG:Ce^3+^, YAGG:Cr^3+^, YAGG:Pr^3+^ phosphors are shown in the Figures S1, S2 of the Supporting Information (S.I.).

All XRPD patterns are constituted by single garnet phase^31^ and are in good agreement with the XRPD pattern of YAGG (PDF#75-0555). The patterns of all phosphors obtained at lower annealing temperatures have broader and less intense peaks due to the lower crystallinity and smaller crystallite sizes. All diffraction peaks became narrower with increasing temperature, which indicates higher crystallinity of the powder in terms of particle size increase or lattice disorder (strain relaxation effect), which could also contribute to the peak broadening. The diffraction patterns were simulated by the Rietveld refinement analysis. For each sample, the agreement between experiment and model was evaluated by R_b_ (4–6%) and the curve of residues. XRPD patterns together with the best fits used for further calculations and the residual plots are shown in the Figures S3–S14 of S.I.. The cell parameter of the garnet phase, the crystallite size and the lattice microstrain are reported in Table 1.

**Table 1:** Cell parameter *a*, crystallite size *D*_XRPD_ and lattice microstrain ε obtained by applying the Rietveld method to the XRPD patterns; specific surface area S_BET_ and particle size (D_BET_, D_TEM_) of the powders.

Sample	T (°C)	a (Å)	D_XRPD_ (nm)	ɛ	S_BET_ (m^2^g^-1^)	D_BET_ (*D_TEM_) (nm)
Undoped YAGG	900	12.192 ± 0.003	52 ± 0.3	0.0023 ± 2	14 ± 2	81
	1000	12.186 ± 0.001	65 ± 0.7	0.0020 ± 1	6 ± 1	189
	1100	12.170 ± 0.001	64 ± 0.2	0.0015 ± 1	6 ± 1	189
	1200	12.165 ± 0.001	102 ± 1.6	0.0010 ± 1	4 ± 1	283
YAGG:Ce^3+^	900	12.190 ± 0.001	73 ± 0.2	0.0030 ± 1	14 ± 2	81
	1000	12.180 ± 0.001	87 ± 0.2	0.0020 ± 1	12 ± 1	94
	1100	12.170 ± 0.001	104 ± 0.1	0.0010 ± 1	9 ± 1	125
	1200	12.165 ± 0.001	335 ± 3.1	0.0010 ± 1	3 ± 1	377
YAGG:Ce^3+^,Cr^3+^,Pr^3+^	900	12.205 ± 0.001	54 ± 0.1	0.0026 ± 2	18 ± 2	63 (50*)
	1000	12.183 ± 0.001	63 ± 0.1	0.0020 ± 1	11 ± 1	103 (> 50*)
	1100	12.175 ± 0.001	127 ± 0.3	0.0010 ± 1	8 ± 1	141 (100–200*)
	1200	12.170 ± 0.001	337 ± 2.8	0.0010 ± 1	2 ± 1	566 (400*)

The cell parameter (*a*) of undoped YAG is 12.008 Å, while those of YGG where all Al^3+^ ions are substituted with gallium is 12.27 Å^40^. The obtained values of cell parameter for the YAGG lattice are due to the difference in ionic radii between Ga^3+^ and Al^3+^ ions (Ga^3+^: 47 pm in tetrahedral site, 62 pm in octahedral site; Al^3+^: 39 pm in tetrahedral site, 53.5 pm in octahedral site)^41^ and thus are in agreement with the hypothesis of formation of YAGG phase having Y:Al:Ga atomic ratio equal to 3:2:3. The cubic cell parameter (*a*) decreases with increasing the annealing temperature was observed even for YAG powders doped with erbium or europium^31,42^ and this behaviour was attributed to the removal of vacancies or impurities^42–46^. It could also depend on a homogeneous disorder introduced into the lattice during the amorphous to crystalline transformation and on an imperfect local stoichiometry. At the same time, the annealing at higher temperature allows a microstrain decrease, indicating a release of this disorder with further densification.

The reticular disorder, described by the microstrain **ε**, varies from 2.3 × 10^–3^ for the 900 °C, to 1.5 × 10^–3^ for the samples obtained at 1000, 1100 and 1200 °C. Knowing that the microstrain is the root mean square of the variations in the lattice parameters across the sample, a higher value means that the distance of the relevant crystal planes is not identical, possibly due to the presence of defects and stress in the lattice. Analogously to what is already well known for long and high temperature treatments, the YAGG crystallites average size increases with temperature increasing.

The cell parameter (*a*) of the doped samples is higher than that of undoped samples prepared at the same temperature. The cell parameter (a), as well as the crystalline size, is higher in the case of the triply doped system in agreement with the partial substitution of Y^3+^ ions by Ce^3+^ and Pr^3+^ and of Al^3+^ ions replaced by Cr^3+^. The ionic radius of Y^3+^ is 102 pm, while the ionic radius of Ce^3+^ and Pr^3+^ are 114 pm and 113 pm, respectively; the ionic radius of Al^3+^ in the B-site is 53.5 pm, lower than the ionic radius of Cr^3+^, 61.5 pm^41^. The increase of crystalline size indicates that doping affects the system at different length scales through different mechanisms: (1) the presence of dopants in the colloidal hydroxides could reduce the energy required for the particle formation, acting as a nucleation site^47^. A lower interfacial surface energy could increase the nucleation rates, which could be compensated by a decreased thermodynamic driving force^48^. (2) The decreased number of nucleation sites can result in a greater number of bigger particles. (3) The interactions between the dopants and surface/grain boundaries affect the surface energy/grain boundary energy, thus, leading the stabilization/destabilisation of the surfaces/grain boundaries, thus, under the same preparation conditions, in presence of dopant more surfaces/grain boundaries can be created; (4) the presence of defects could create a charge imbalance, which can be compensated for by the creation of oxygen vacancies. This in turns prompts a larger oxygen ion motion and thus increased grain growth and bigger particle size. Understanding the mechanism needs further study. Since it is not within the scope of this paper, we did not pursue an investigation.

In all cases, as observed in the undoped samples, the size increases with the temperature increase. In presence of dopants, together with the reason already discussed for undoped samples, the microstrain decrease could also indicate a segregation of dopant ions to the surface of garnet domains^42^.

**Figure 2:**
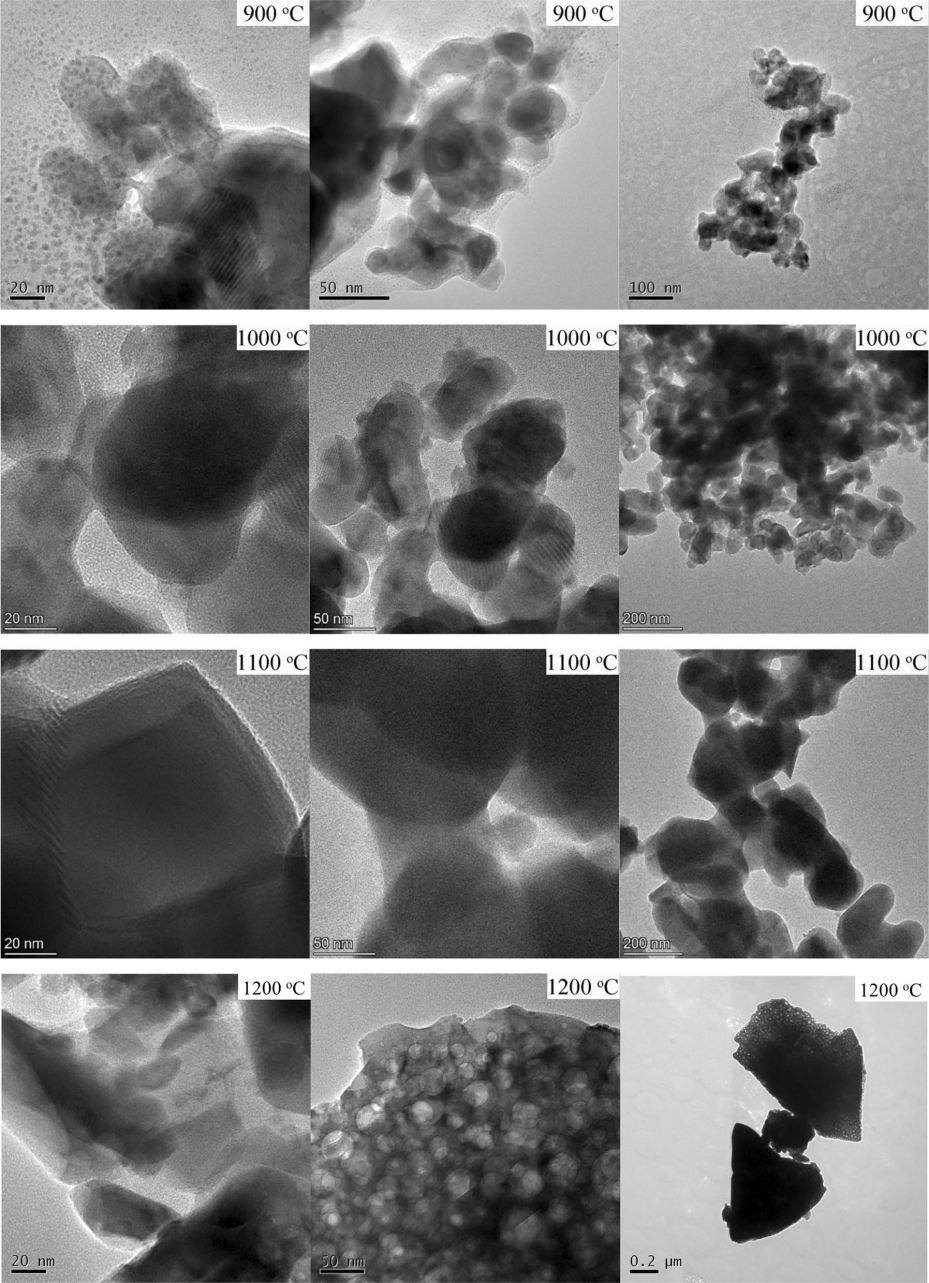
TEM micrographs at different magnifications of the YAGG:Ce^3+^,Cr^3+^,Pr^3+^ nanophosphors annealed at different temperatures.

All the nitrogen adsorption–desorption isotherms are type IV according to IUPAC terminology. In all sets of samples, the S_BET_ decreases with the temperature of treatment (Table 1), indicating the increase of particle size, which, compared with the D_XRPD,_ is an indication of the low grade of agglomeration^39,49,50^. Thus, the size of aggregates evaluated by the D_BET_ value particles for the powder annealed at 900 °C is still in the same order of magnitude as the average individual D_XRPD_. While above this annealing temperature, the size of aggregates is ~ 3 times larger, which confirms, that creating the solid bridges between the particles starts above these temperatures. Considering that the D_XRPD_ increase and the microstrain decrease lead to the particle growth size, and considering the obtained values of D_BET_, the TEM micrographs of YAGG:Ce^3+^,Cr^3+^,Pr^3+^ samples were acquired. Three TEM micrographs with different magnifications of YAGG:Ce^3+^,Cr^3+^,Pr^3+^ samples annealed at different temperatures are reported in Fig. 2. From TEM micrographs it can be deduced that particles treated at 900 °C have slightly irregular shape, sizes around 50 nm (confirming D_XRPD_ diameters in primary nanoparticles) and are agglomerated (confirming the D_BET_ values of secondary nanoparticles). The particle sizes as well as agglomeration degree increase with increasing temperature. The higher temperature facilitates the rapid arrangement of the crystalline structure and subsequent coalescence of the chemical species to form particle agglomeration. This is more evident in the sample annealed at 1200 °C, where a very big agglomeration and coalescence of primary particles occurred. The contrasted bands consist of pairs of Fresnel fringes (black/white or white/black) that occur when primary nanoparticles favourably overlap. These fringes extended to the entire nanoparticle, thus revealing the interfaces with neighbouring nanoparticles. We can assess the presence of numerous nano-crystalline domains extended to the entire nanoparticle volume.

The EDS spectrum of one single grain of sample treated at 1100 °C, was collected obtaining the formula Y_3.09_Al_2_Ga_2.72_O_10.36_. This is very close to the theoretical one: Y_3_Al_2_Ga_3_O_12_, after elaboration on Raw EDS spectrum setting the Carbon as "deconvolution only" and using the Schreiber–Wims model for the ionization cross-section correction^51^ This finding is a validation of the methodology of the synthesis to guarantee the expected stoichiometry. The Raw EDS data are reported in the Figure S15 of the S.I..

### Optical properties

**Figure 3:**
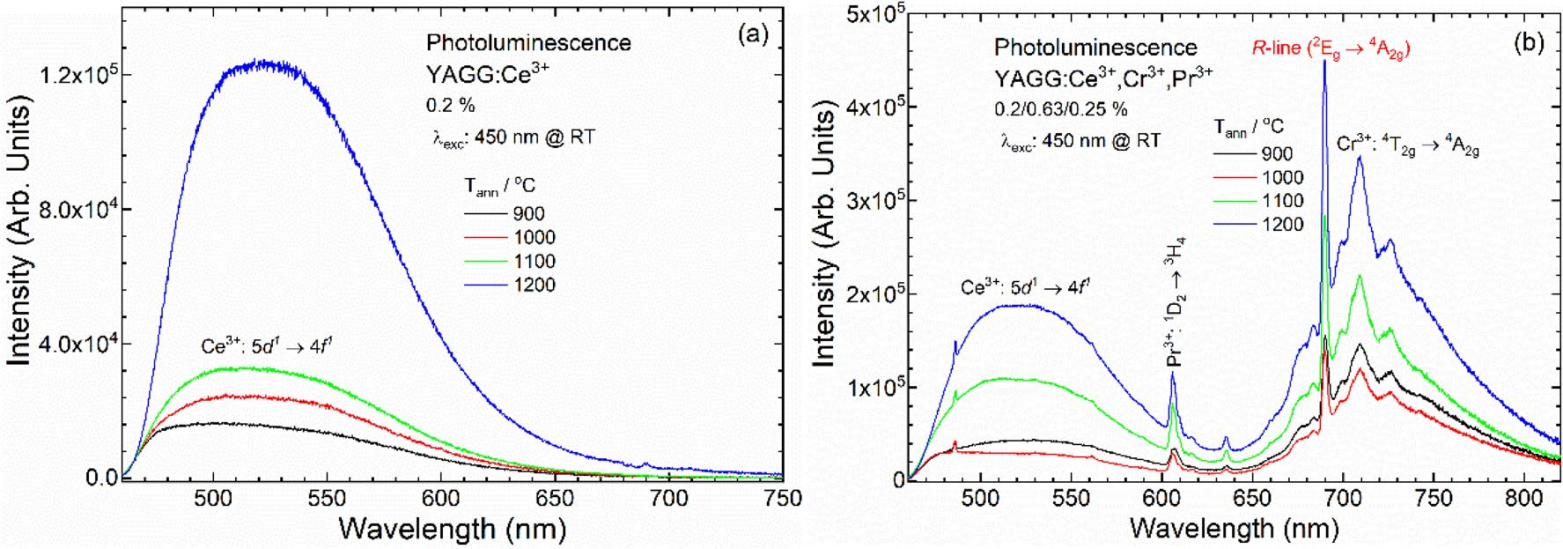
PL spectra of (**a**) YAGG:Ce^3+^ and (**b**) YAGG:Ce^3+^,Cr^3+^,Pr^3+^ nanophosphors annealed at different temperatures. λ_exc_: 450 nm at RT.

PL and PLE spectra were analysed in order to investigate the optical properties of doped samples. The PL spectra of YAGG:Ce^3+^ and YAGG:Ce^3+^,Cr^3+^,Pr^3+^, annealed at different temperatures, were detected upon excitation at 450 nm (Fig. 3). The increase of the luminescence intensity of YAGG:Ce^3+^ and of YAGG:Ce^3+^,Cr^3+^,Pr^3+^ nanophosphors with increasing annealing temperature was observed and is associated with the particle size increase^45,46^. The phosphors with large particle size have low specific surface area, and thus have less surface defects^52^. The same tendency was observed for PLE spectra that were detected for each ion (S.I. Fig. S16–S19). As we can see in Fig. 3, the best sample in terms of PL properties, is the one annealed at 1200 °C, even if PersL intensity for this sample is lower than the PersL for sample annealed at 1100 °C (Fig. 7d). In addition, the particle size of the sample annealed at 1200 °C is no longer nanometric but sub-micron size (337 nm, see Table 1), being ~ 3 times larger than the sample annealed at 1100 °C (127 nm). For some practical applications such as bio-imaging, the nano-sized phosphors show greater potential than the micro-sized phosphors^1,5^. The YAGG:Ce^3+^,Cr^3+^,Pr^3+^ nanophosphor annealed at 1100 °C shows good PL properties, and the particle size is around 100 nm. Comprehensively considering the particle size and luminescence intensity, 1100 °C is the optimal annealing temperature. The next discussion is focused on this sample, comparing its spectroscopic properties to the singly doped with Ce^3+^ sample. Also, the PersL decay curves (S.I. Fig. S23), registered for the samples annealed at 1100 and 1200 °C, show the similar emission intensities and durations.

**Figure 4:**
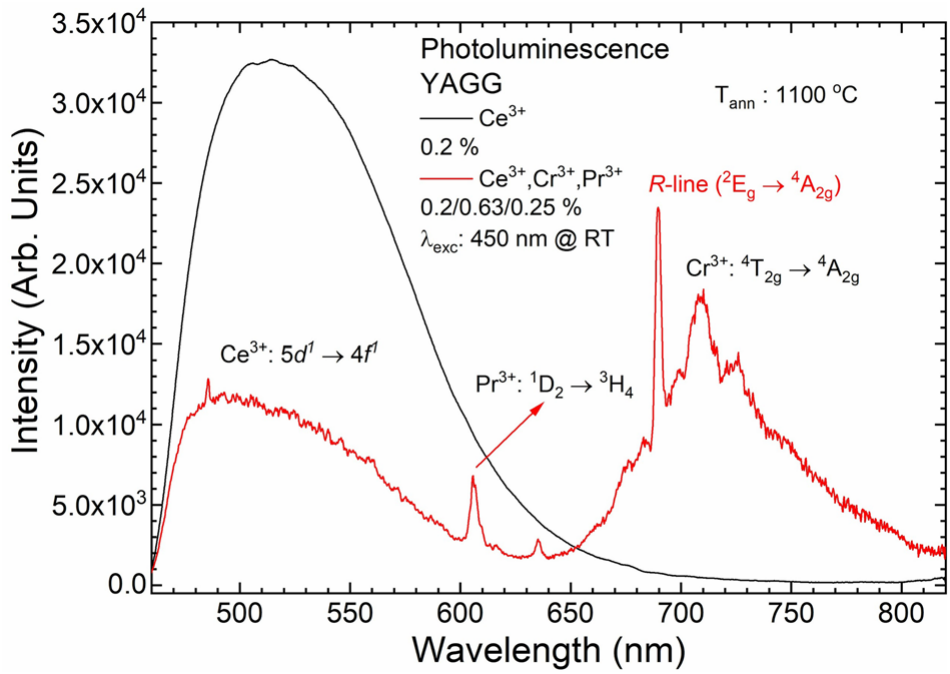
PL of YAGG:Ce^3+^ and YAGG:Ce^3+^,Cr^3+^,Pr^3+^ nanophosphors annealed at 1100 °C. λ_exc_: 450 nm at RT.

The PL spectra of YAGG:Ce^3+^ and YAGG:Ce^3+^,Cr^3+^,Pr^3+^, annealed at 1100 °C, were collected upon excitation at 450 nm (Fig. 4). The typical broadband Ce^3+^ emission (centred at 520 nm) due to the 5*d* → 4*f* transition for YAGG:Ce^3+^ and YAGG:Ce^3+^,Cr^3+^,Pr^3+^ nanophosphors was observed. For the triply doped YAGG:Ce^3+^,Cr^3+^,Pr^3+^ sample, in addition to the Ce^3+^ emission, the broad band with maximum around 709 nm (Cr^3+^:^4^T_2g_ → ^4^A_2g_ transition) and the strong narrow lines due to 4*f* → 4*f* transitions in Pr^3+^:^1^D_2_ → ^3^H_4_ (606 nm) and ^3^P_0_ → ^3^F_2_ (634 nm) were detected. The weak sharp lines at 485, 560 and 616 nm due to *f–f* transitions in Pr^3+^:^3^P_0_ → ^3^H_4_; ^3^H_5_ and ^3^H_6_, respectively^53^ were also observed.

Comparing the PL spectra (Fig. 4) of YAGG:Ce^3+^ and YAGG:Ce^3+^, Cr^3+^, Pr^3+^, it was found that the intensity of Ce^3+^ emission decreased by more than 30% for the triply doped sample. This difference can be connected to re-absorption of Ce^3+^ emission by other doping ions. As result, two parallel processes can be observed together: ET and re-absorption by Cr^3+^.

**Figure 5:**
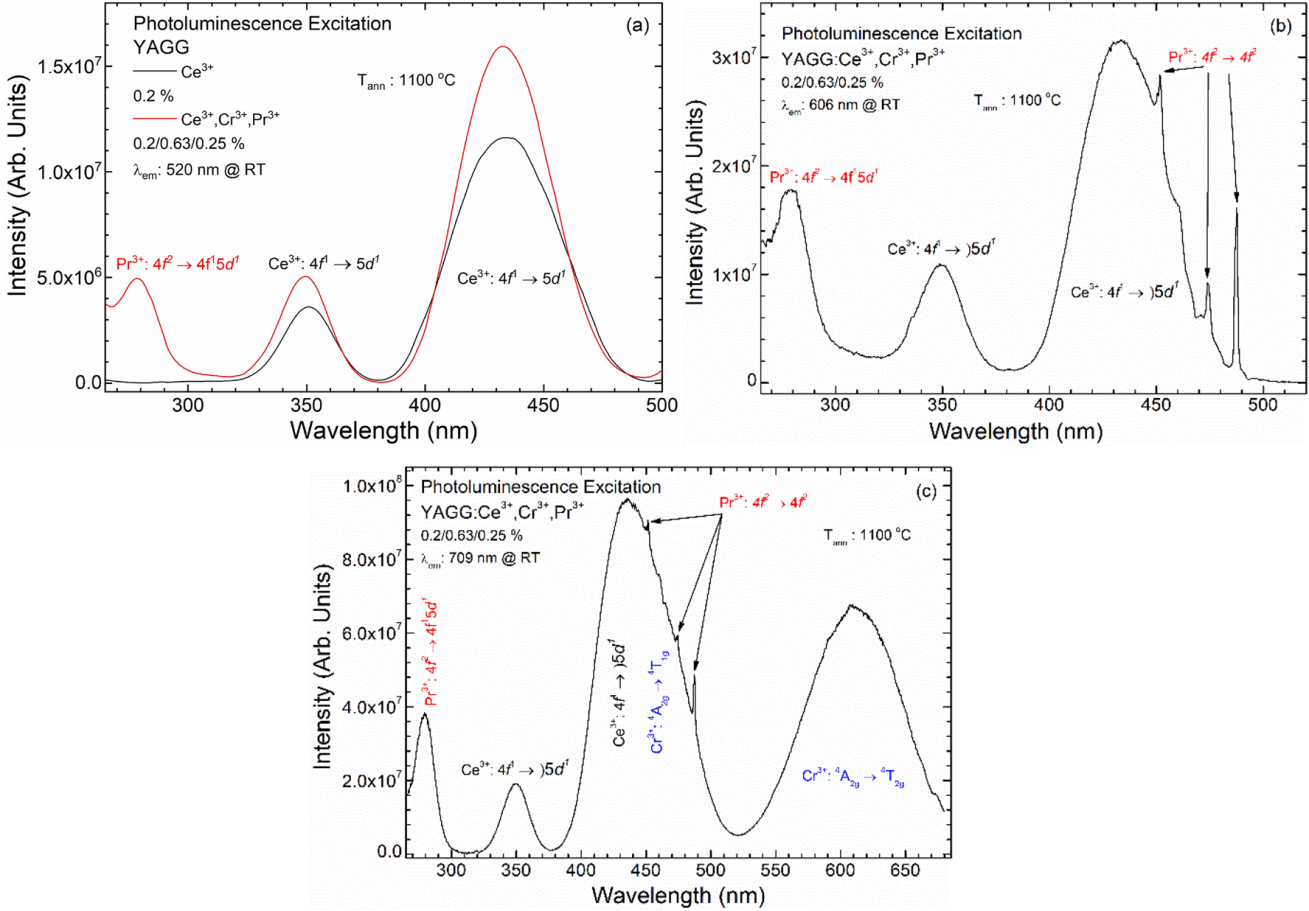
PLE spectra of singly and triply doped YAGG annealed at 1100 °C. Monitoring the emission wavelength at: (**a**) 520, (**b**) 606, (**c**) 709 nm at RT.

The PLE spectra were collected at 520 (5a), 606 (5b) and 709 nm (5c), for Ce^3+^, Pr^3+^ and Cr^3+^ emission, respectively (Fig. 5). PLE spectra for each excitation, as *f*(T), were reported in the Figures S16–S19 of the S.I. In all spectra, broad bands centred at 350 and 435 nm were recorded, while the band at 278 nm appeared only for the co-doped sample. For the excitation spectra of Pr^3+^ (at 606 nm) and Cr^3+^ (at 709 nm) the sharp bands near 450 nm are due to 4*f → *4*f* transition of Pr^3+^. The presence of Pr^3+^ bands on the Cr^3+^ PLE spectra indicates the presence of ET from Pr^3+^ to Cr^3+^ ions. In the PLE spectra of Cr^3+^ (at 709 nm) a broad band at 617 nm (Cr^3+^:^4^A_2g_ → ^4^T_2g_ transition) was also observed.

In the PLE spectra of Ce^3+^, acquired at 520 nm, the two bands centred at 350 and 435 nm are attributed to the transition from 4*f* to *5d* levels, split by the crystal field. Broad band at 278 nm, which appeared for samples YAGG:Ce^3+^,Cr^3+^,Pr^3+^ and which is absent for the YAGG:Ce^3+^, connected with Pr^3+^:4*f* → 5*d* transitions^11^ as well as with Cr^3+^:^4^A_2g_ → ^4^T_1g_(^4^P) transition^54,55^, which overlap.

In the PLE spectra of Pr^3+^, acquired at 606 nm, the broad band recorded at 278 nm corresponds to Pr^3+^:4*f* → 5*d* transitions and sharp bands near 450 nm correspond to Pr^3+^:4*f* → 4*f* (^3^H_4_ → ^3^P_J_) transitions. The narrow peaks (Pr^3+^:4*f* → 4*f*) are in superposition with the broad band centred at 435 nm coming from Ce^3+^ and the existence of Ce^3+^ bands in Pr^3+^ PLE spectra can indicate the ET from Ce^3+^ to Pr^3+^ ions^11^.

The PLE spectra of Cr^3+^ can be ascribed to the Cr^3+^ ion absorption corresponding to spin-allowed transitions:^4^A_2g_ → ^4^T_2g_ (maximum at 617 nm) and ^4^A_2g_ → ^4^T_1g_ (maximum at 445 nm)^54,55^. It should also be noted that a broad band with maximum at 445 nm can be a superposition of two bands: Cr^3+^:^4^A_2g_ → ^4^T_1g_ transition and another spin-allowed Ce^3+^ 4*f* → 5*d* transition. The effect of overlapping two separate bands is most pronounced at lower annealing temperatures, because the distance between them is greatest. As the annealing temperature increases (and also the crystallite size), the crystal field strength changes and the Cr^3+^:^4^A_2g_ → ^4^T_1g_ transition band shifts to the blue region^56^ and, for a temperature of 1200 °C, completely overlaps with the Ce^3+^ 4*f* → 5*d* transition band. The band centred at 350 nm from Ce^3+^ may also indicate the ET from Ce^3+^ to Cr^3+^^21^. The band located at 278 nm (as we noted for Ce^3+^ excitation spectra) may be a result of superposition of Cr^3+^:^4^A_2g_ → ^4^T_1g_(^4^P)^55^ and Pr^3+^:4*f* → 5*d* transitions^53^. Additionally, the series of sharp bands at around 450 nm which refer to Pr^3+^:4*f* → 4*f* (^3^H_4_ → ^3^P_J_) transitions are also presented^11,53^.

**Figure 6:**
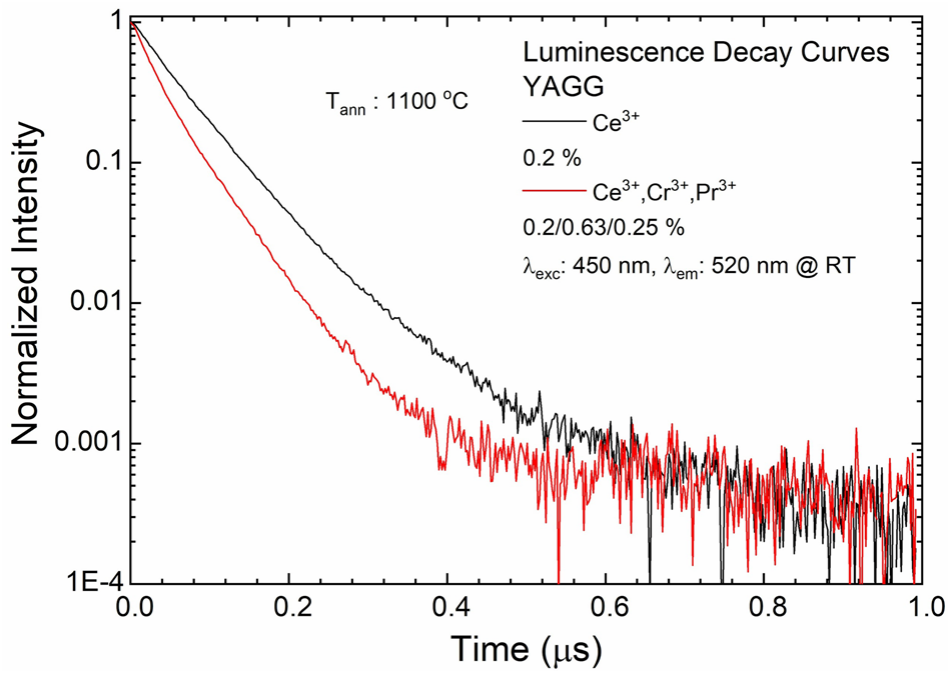
The luminescence decay curves of YAGG:Ce^3+^ and YAGG:Ce^3+^,Cr^3+^,Pr^3+^ nanophosphors annealed at 1100 °C. λ_exc_: 450 nm and λ_em_: 520 nm at RT.

The emission decay time (DT) of the YAGG:Ce^3+^ and the YAGG:Ce^3+^,Cr^3+^,Pr^3+^ samples were measured for the emission of Ce^3+^ at 520 nm under the laser excitation at 450 nm. The data acquired for the samples annealed at 1100 °C are reported in Fig. 6. The DT for the co-doped samples are not the mono exponential ones. The main factor that can influence the obtained data are the appearance of afterglow for the co-doped crystal, which significantly increases the background. The effective DT^57^ was calculated to compare the obtained curve shapes and further calculate the ET coefficient. Based on the DT, the ET efficiencies from Ce^3+^ to Pr^3+^ and Cr^3+^ were calculated for each annealing temperature (see S.I. Fig. S20). The DT decrease with increasing annealing temperatures could be explained by an increase of grain size of nanophosphors^45^.

The effective DT of the YAGG:Ce^3+^ and YAGG:Ce^3+^,Cr^3+^,Pr^3+^ nanophosphors are equal at 69 and 52 ns, respectively. The value of DT decreases for the phosphors co-doped with Cr^3+^ and Pr^3+^ is caused by ET from excited states of Ce^3+^ to Cr^3+^ and/or Pr^3+^. The efficiency of ET from Ce^3+^ to Pr^3+^ and Cr^3+^ is equal to 24%. This is close to value for YAGG:Ce^3+^,Cr^3+^ with similar concentrations of Ce^3+^ and Cr^3+^ ^58^.

The QY are equal to 47% and 27%, respectively for singly and triply doped nanophosphors. The QY decreasing in the latter case may result from trapping energy. For this reason, the PersL properties of the YAGG:Ce^3+^,Cr^3+^,Pr^3+^ and of YAGG:Ce^3+^ nanophosphors were also investigated.

### Persistent luminescence properties

**Figure 7:**
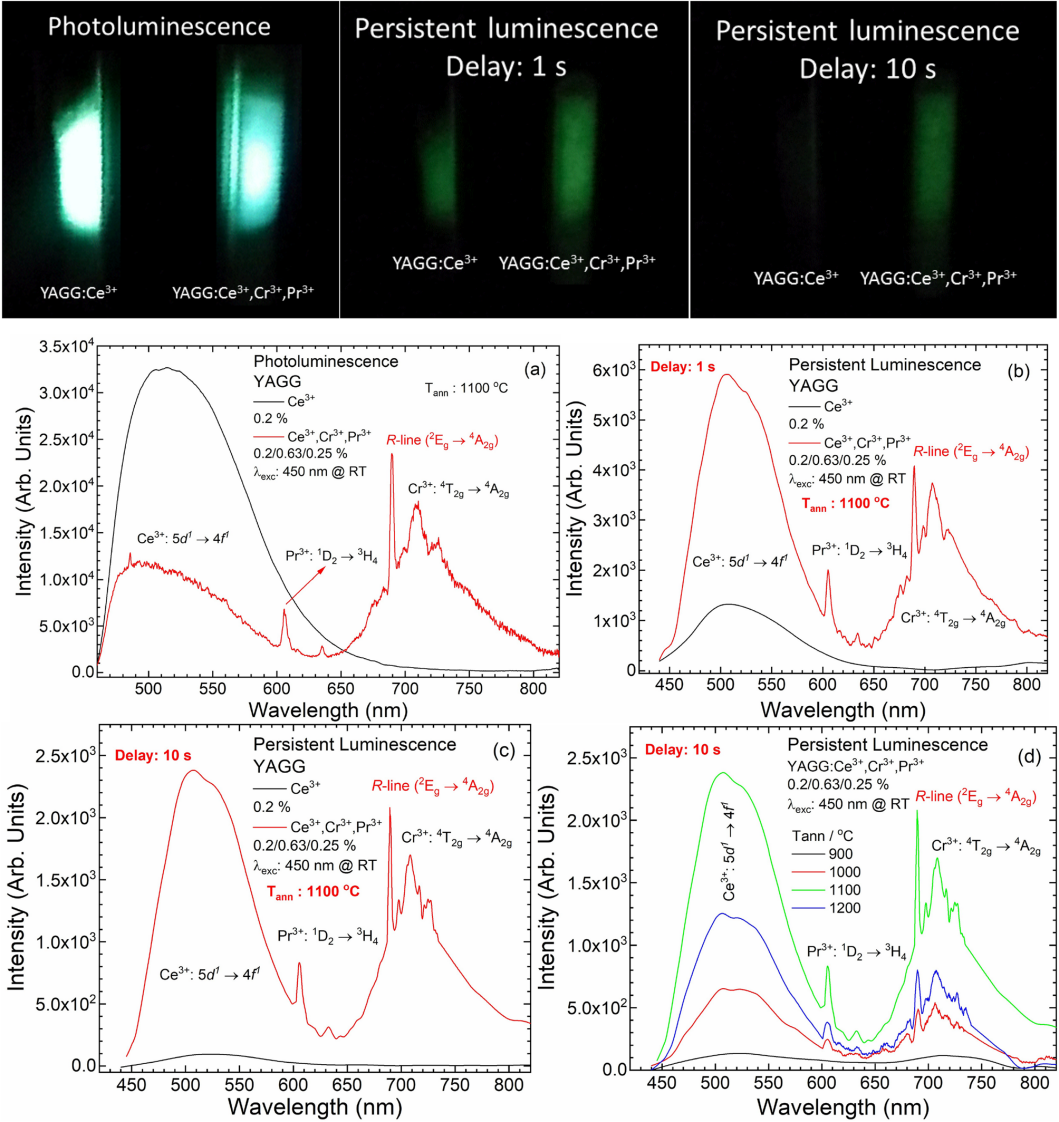
Photos of PL and PersL, as well as PL (**a**) and PersL spectra of YAGG:Ce^3+^ and YAGG:Ce^3+^,Cr^3+^,Pr^3+^ nanophosphors annealed at 1100 °C after 1 s (**b**) and 10 s of delay (**c**) and PersL spectra as a function of particle size (**d**).

The PersL spectra of the YAGG:Ce^3+^ and YAGG:Ce^3+^,Cr^3+^,Pr^3+^ nanophosphors after blue light (450 nm) irradiation were recorded as a function of the nanophosphors’ size (annealing temperature), and are presented in Fig. 7. It can be seen that the PL and PersL spectra of both nanophosphors show similar shape bands. Both spectra are composed of two broad bands from Ce^3+^ and Cr^3+^ and sharp lines from Pr^3+^. The main broad band centred at 520 nm is ascribed to the 5*d* → 4*f* electronic transition of Ce^3+^, the broad band centred at around 709 nm is due to the ^4^T_2g_ → ^4^A_2g_ transition of Cr^3+^. Finally, the sharp line at 606 nm is ascribed to the ^1^D_2_ → ^3^H_4_ transition of Pr^3+^. Only the intensity ratio between associated Ce^3+^ and Cr^3+^ bands are changing analogously as we previously observed for YAGG:Ce^3+^,Cr^3+^^59^. The PersL intensity (Fig. 7d) increases with increasing annealing temperature up to 1100 °C. A further increase in the annealing temperature to 1200 °C causes a PersL decrease, probably due to defect elimination, which may work as traps and lead to PersL.

The photographs and corresponding PL and PersL spectra of singly and triply doped YAGG nanophosphors, acquired under excitation at 450 nm, at 1 and 10 s after ceasing irradiation source are presented in Fig. 7. The PL CIE coordinates of YAGG:Ce^3+^,Cr^3+^,Pr^3+^ annealed at 1100 °C were calculated. The results are (0.34, 0.54). Additionally, the CIE coordinates of PersL with delay time 1 and 10 s were also calculated. The results are 1 s: (0.28, 0.50) and 10 s: (0.28, 0.52). The CIE diagram was added in the Supplementary Information (Fig. S21). The YAGG:Ce^3+^,Cr^3+^,Pr^3+^ shows a yellow green component PL in the CIE coordinates. Compared to the PL, the PersL (recorded both after at 1 and 10 s) slightly shifts to green. The reason is that the red or the NIR PersL of Cr^3+^ and Pr^3+^ is caused by the energy transfer from Ce^3+^. The energy transfer is less efficient than the excitation direct from the laser diode. The red emission from the YAGG:Ce^3+^,Cr^3+^,Pr^3+^ cannot be observed by naked eye, because the red emission from Cr^3+^ and Pr^3+^ is weaker than the green emission from Ce^3+^, and the maximum of spectral sensitivity of the human eye is in the green colour region.

**Figure 8:**
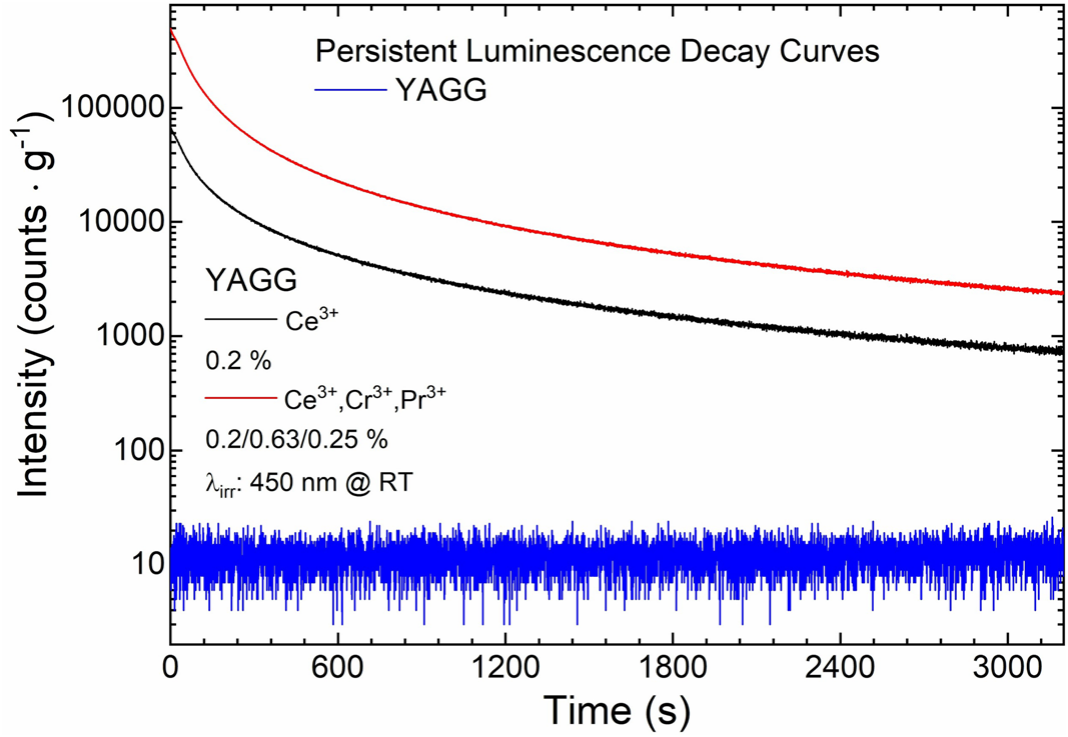
PersL decay curves of YAGG:Ce^3+^ and YAGG:Ce^3+^,Cr^3+^,Pr^3+^ nanophosphors. λ_irr_: 450 nm (1 mW cm^−2^) for 5 min at RT and the observed signal intensity was corrected by mass.

One of the side effects of co-doping YAGG:Ce^3+^ nanophosphors with Cr^3+^ and Pr^3+^ ions is extending the duration of the PersL. Comparing photographs taken immediately after ceasing the excitation source and after 10 s, it is possible to observe that YAGG:Ce^3+^ has a very intense PL but after removing excitation source, emission quickly disappears. On the other hand, YAGG:Ce^3+^,Cr^3+^,Pr^3+^ nanophosphors have weaker PL (compared to corresponding YAGG:Ce^3+^) but the intensity of the signal remains almost unchanged after the removal of the excitation source (Fig. 8). The PersL decay curve was recorded in the visible region (from 340 to 850 nm) for each sample (Fig. 8; SI Fig. S22–S23) after irradiation with LD 450 nm (1 mW cm^−2^) for 5 min, and the observed signal intensity was corrected by mass. As a reference signal, the PersL decay curve from the undoped YAGG was recorded at the same condition. This approach was possible because the measurements were performed under the same conditions for similar samples. Unfortunately, because of limits of equipment used for TL measurements, we cannot directly compare the duration time of PersL for obtained nanophosphors with other existing analogues, as it was proposed and described e.g. by Smet et al.^60^. As it is well evidenced by the obtained curve, the PersL is longer than 1 h (according to the obtained data). The green emission of YAGG:Ce^3+^,Cr^3+^,Pr^3+^ is easy to be recognized up to 30 min in a dark room with the naked eyes.

### Thermoluminescence

**Figure 9:**
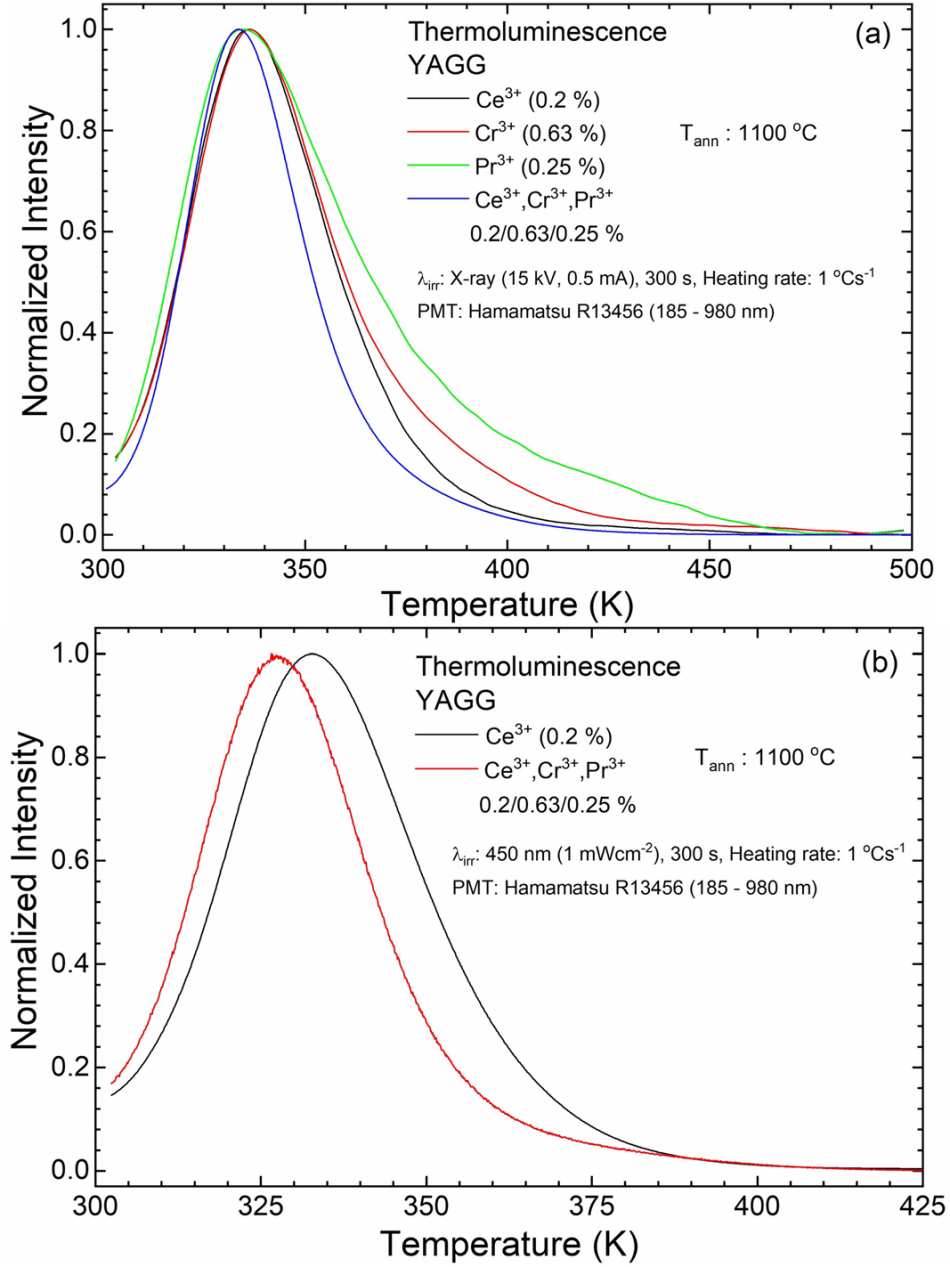
Normalized TL glow curve of singly and triply doped YAGG nanophosphors annealed at 1100 °C. (**a**) λ_irr_: X-ray (15 kV, 0.5 mA), (**b**) λ_irr_: 450 nm (1 mW cm^−2^) for 5 min at RT.

**Figure 10:**
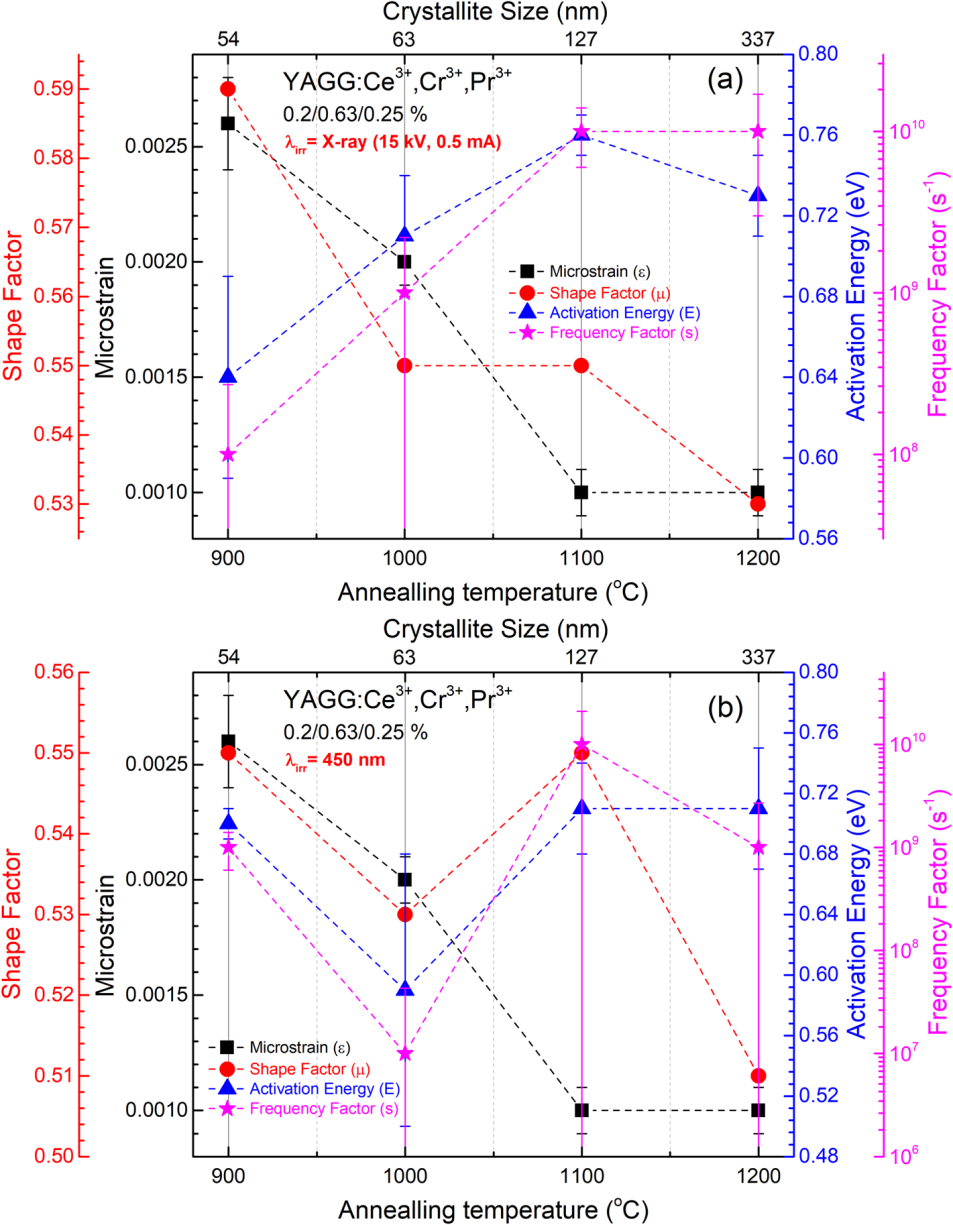
Correlation between microstrain and TL parameters (shape factor, activation energy, and frequency factor) for the X-ray (**a**) and blue-light (**b**) irradiation as a function of the crystallite size (annealing temperature).

Glow curves of singly doped YAGG:Ce^3+^, YAGG:Cr^3+^ and YAGG:Pr^3+^ and triply doped YAGG:Ce^3+^,Cr^3+^,Pr^3+^ after irradiation by blue LD (@450 nm) and X-ray source were recorded and analysed to determine shape factors (*μ*) and order of kinetics (*b*) by using the methods of analysis based on the shape of the glow curve^61,62^. Each curve was detected after the sample was illuminated for 5 min, and the heating rate was 1 °C s^−1^ (heating rate *β*—0.25, 0.50, 0.75, 1.0 and 2.0 °C s^−1^ were also used for calculation of *E* and *s* by heating rate method^61^). As it is evident (Fig. 9 and other annealing temperatures in the S.I.), all curves show a broad band that can be the result of the quasi-continuous trap distribution producing a broad band centred at around 335 K. From TL curves of singly doped nanophosphors annealed at 900 °C, we can distinguish a series of separated traps with different trap depths. As the annealing temperature increases, the specific surface area and the number of surface defects decreases (as O–H groups on the surface^63,64^). As a consequence, the number of shallow traps decreases. For higher annealing temperatures, the defects mainly coming from the partial substitution of Y^3+^ ions by Ce^3+^ and Pr^3+^ and of Al^3+^ ions replaced by Cr^3+^ begin to act as traps. At the same time, it has to be noted that for annealing temperature at 1200 °C, the shape of the line in the high temperature side of the TL peak approaches the curve with second order. As can be seen from Table 1, *ε* for annealed nanoparticles at 1100 °C and 1200 °C is unchanged, which means that a further increase in the size does not reduce the lattice microstrain, while the cell parameter is further decreased. This means that the ordering of the ions within the crystal lattice improves and, as a result, the intensity of PL further increases reaching the maximum for the sample annealed at 1200 °C. Moreover, the luminescence intensity increases and its QY is an indication of the elimination of surface defects, which is clearly seen from the above data. The disappearance of deeper traps thus reduces the PersL duration. That is, the resulting sample has much brighter initial (first 10 s) emission but shorter duration. The addition of Cr^3+^ forms stable med-depth traps which, as can be seen from the TL curves of single-doped Cr^3+^, remain unchanged for all annealing temperatures (Fig. 10; S.I. Table S1). As we can see, the TL curves of samples singly doped with Ce^3+^ after X-ray irradiation have a different shape on the high-temperature side compared to the curves after LD (@450 nm) irradiation. This may be due to the fact that, by using the high energy X-rays, we fill deeper traps with maximum around 400 K. For the blue light irradiation, no signal from Cr^3+^ and Pr^3+^ singly doped samples was detected, because the energy of blue light is insufficient to prompt the electrons of Cr^3+^ and Pr^3+^ to the conduction band. On the other hand, for the Ce ion-doped sample, the movement of electrons from the excited 5d level to the electron traps passes through the conduction band. After the X-ray irradiation, TL curves of YAGG:Cr^3+^ annealed at different temperatures show similar shape and position of the maximum, while some differences have been observed for YAGG:Ce^3+^ and YAGG:Pr^3+^. The TL curves shift to higher temperature for YAGG:Ce^3+^, while the curves of YAGG:Pr^3+^ shift to lower temperature.

Based on the estimation of trap depth for materials exhibiting PersL, the optimal value of trap depth may be in the range 0.58–1.13 eV, while the *s* value lies in the range 10^8^–10^14^ s^−1^. In particular, at room temperature (around 300 K) and activation energy around 0.71 eV (same as we have for triply doped samples) for long term PersL *s* should be around 10^10^, while for *s* equal 10^8^, the best PersL will be at about 350 K^65^. An additional factor that positively affects the duration of afterglow is its order. The TL peak must follow second-order kinetics. For values of *E* less than the specified one, together with the decrease of the parameter *s* and the order of the curve, the recombination process will take place quickly. As a result, the PersL duration will be shorter. As we can see from Table S1, *μ* is in the range between 0.51 and 0.72, which indicates that it is a second (*μ* = 0.52) or more order TL peak, responsible for overlapping at least two or more traps with shallow and deep depth resulting in the long PersL. The *E* of singly doped YAGG:Ce^3+^, YAGG:Cr^3+^and YAGG:Pr^3+^ and triply doped YAGG:Ce^3+^,Cr^3+^,Pr^3+^ nanophosphors is found around 0.71 eV (with exception around 0.5 eV for the sample singly doped with Ce^3+^ ions) which is in the range for which one can expect a long PersL at room temperature^65^. As for the **s** factor, it acquires optimal values in relation to *E* for samples prepared at 1100 °C^65^. Consequently, the bright initial emission for samples prepared at higher temperature (1200 °C) and without co-dopants (at least Cr^3+^) was observed after the removal of the irradiation source, but the duration was significantly shorter than the samples annealed at 1100 °C and co-doped with Cr^3+^. It means that, at almost the same activation energy for samples prepared at 1100 °C and 1200 °C, we register a more homogeneous distribution of traps (the order of the TL peak follows the first-order) and the probability of electron release increases with its subsequent recombination. The latter may be, due to the fact, that for the samples prepared at 1200 °C Ce^3+^ ions are distributed more evenly on the crystal lattice and the probability of its finding near the trap increases.

## Conclusions

This study shows the correlation occurring between the microstructure, the particle size and PersL properties. It was confirmed that the optical properties of YAGG:Ce^3+^,Cr^3+^,Pr^3+^ nanophosphors are significantly affected by the annealing temperature. By showing a lower degree of agglomeration and a smaller size of nanoparticles, samples annealed at 1100 °C show at the same time a very effective ET from Ce^3+^ to Cr^3+^ and Pr^3+^ compared to samples annealed at 1200 °C. On the other hand, smaller crystallites obtained below 1100 °C have more surface defects which can lead to luminescence quenching. The same trend was observed for the PersL where the duration time of emission of samples annealed at 1100 °C was much longer than the samples annealed at lower temperatures. It should be noted, however, that for samples annealed at 1200 °C PersL duration and intensity was almost the same as the recorded samples annealed at 1100 °C. The observed changes in ET from Ce^3+^ to Cr^3+^ and Pr^3+^ can be correlated with the difference of the lattice parameter. Lattice parameter in turn affects the crystal field strength and the Cr^3+^:^4^A_2g_ → ^4^T_1g_ transition band completely overlaps with the Ce^3+^:4*f* → 5d transition band for annealing temperature 1200 °C. Additionally, a higher annealing temperature and ordered surface structure does not lead to a significant improvement in both activation energy *E* and frequency factor *s* (*E* = 0.73 eV, *s* = 1.18 × 10^10^ s^−1^ for 1200 °C vs *E* = 0.76 eV, *s* = 2.38 × 10^10^ s^−1^ for 1100 °C—after X-ray irradiation; *E* = 0.71 eV, *s* = 5.55 × 10^9^ s^−1^ for 1200 °C vs *E* = 0.71 eV, *s* = 5.56 × 10^9^ s^−1^ 1100 °C—after blue LD irradiation). However, for the samples annealed at 1200 °C, the high-temperature shoulder disappears on the TL curves, compared to samples annealed at 1100 °C. Thus, based on the activation energy in relation to the *s* factor, we proved that we are able to point-out the material characterized by the best PersL properties even without measurements of the absolute intensity (as it is in our case for comparison of the samples obtained at 1100 and 1200 °C, indicating that PersL for the sample annealed at 1100 °C is better at RT).

Summarizing, the co-precipitation can be considered as a low-cost method for the production of triply doped tailored PersL nanophosphors, which may be used for the fabrication of optical ceramics, polymeric composites and silica-based thin films. It can be concluded that higher annealing temperature in the tested range did not improve the optical properties of nanophosphors. Therefore, the YAGG nanophosphor annealed at 1100 °C showing an acceptable degree of particle agglomeration may already be suitable for practical applications. The reported stable emissions from Cr^3+^ can have a significant potential application in biological imaging technology in the first biological window. The emission from Pr^3+^ indicates the nanophosphors have potential to work as luminescent concentrators in band-matched solar cells, although the efficiency of PersL nanophosphors must be still further optimised in particular by controlling their synthesis and thermal treatment conditions.

## Supplementary Information

Supplementary Information
