# Supplementary material for: Particle size-related limitations of persistent phosphors based on the doped Y3Al2Ga3O12 system
Source: Sci Rep. 2021 Jan 8;11:141. doi: 10.1038/s41598-020-80335-9 (PMC7794296; doi:10.1038/s41598-020-80335-9)
Supplement: Supplementary file 1 — Supplementary Information [file 41598_2020_80335_MOESM1_ESM.docx]

**Supplementary Information**

**Particle size-related limitations of persistent phosphors based on the doped Y_3_Al_2_Ga_3_O_12_ system**

*Vitalii Boiko^a^, Marta Markowska^a^, Zhengfa Dai^a^, Cristina Leonelli^b^, Cecilia Mortalò^c^, Francesco Armetta^d^, Federica Ursi^d^, Maria Luisa Saladino^d*^, Dariusz Hreniak^a^*


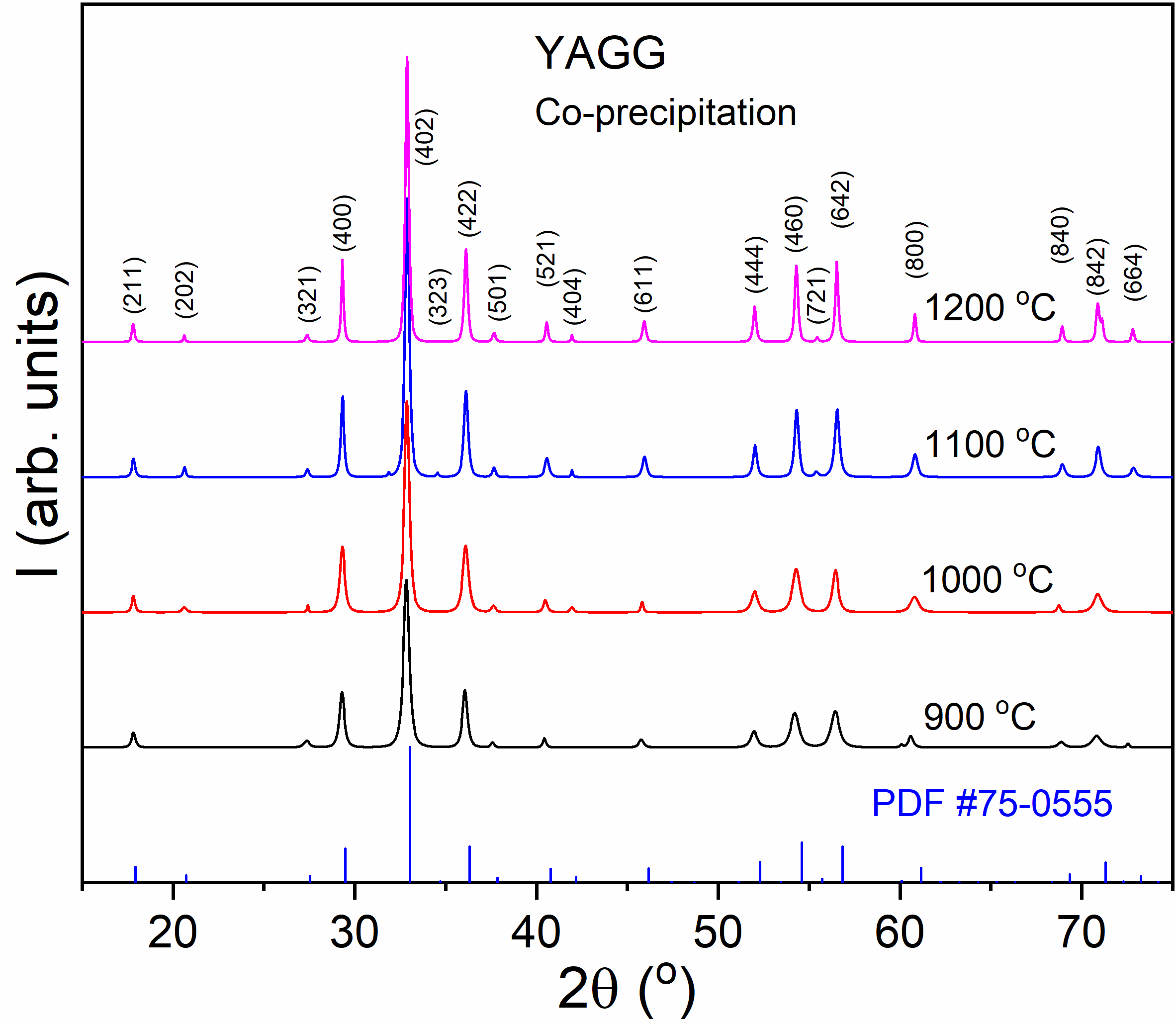


Figure S1. XRPD patterns of YAGG:Ce^3+^,Cr^3+^,Pr^3+^ annealed at different temperatures in air.









Figure S2. XRPD patterns of (a)YAGG:Ce^3+^, (b) YAGG:Cr^3+^ and (c) YAGG:Pr^3+^ annealed at different temperatures in air.


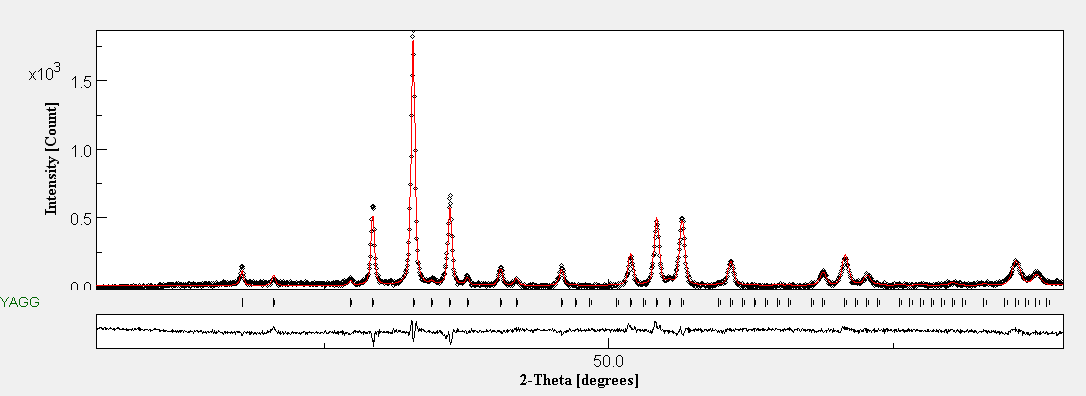


Figure S3. XRD patterns (dots) and the Rietveld fits (full lines) of the YAGG sample obtained at 900° C for 1 h. Bar sequences of YAGG reference diffraction patterns and residual plot are shown along the bottom.


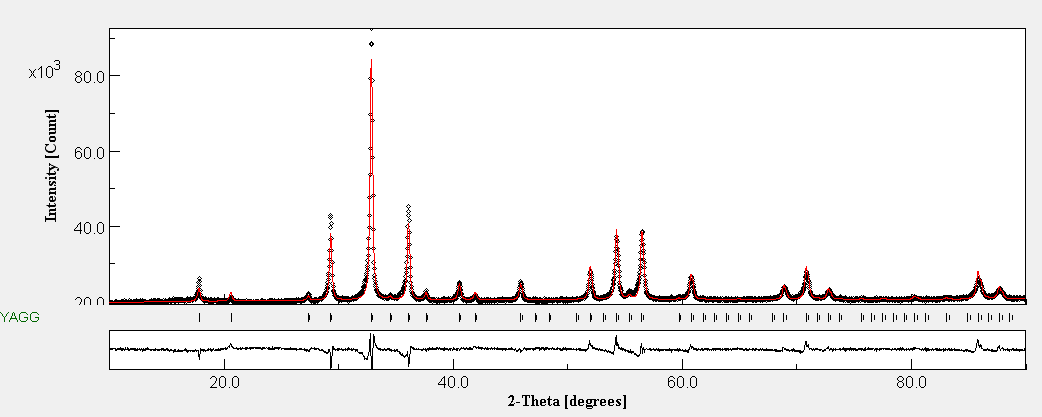


Figure S4. XRD patterns (dots) and the Rietveld fits (full lines) of the YAGG sample obtained at 1000° C for 1 h. Bar sequences of YAGG reference diffraction patterns and residual plot are shown along the bottom.


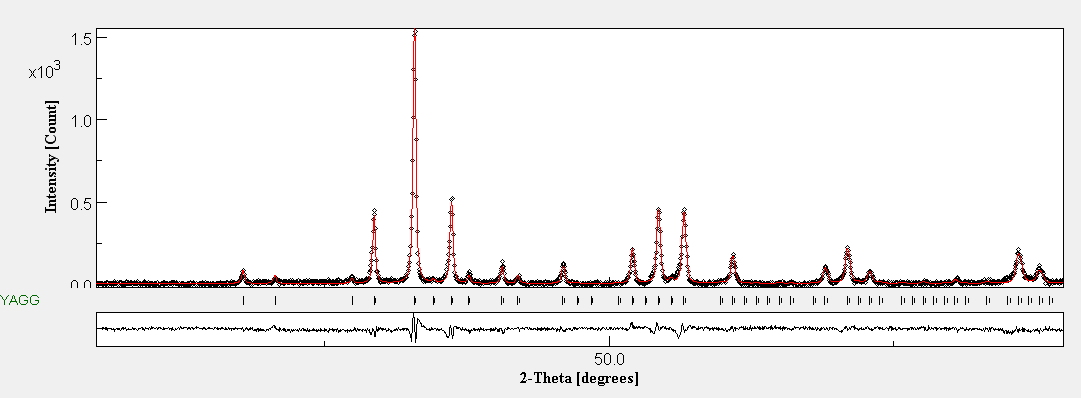


Figure S5. XRD patterns (dots) and the Rietveld fits (full lines) of the YAGG sample obtained at 1100° C for 1 h. Bar sequences of YAGG reference diffraction patterns and residual plot are shown along the bottom.


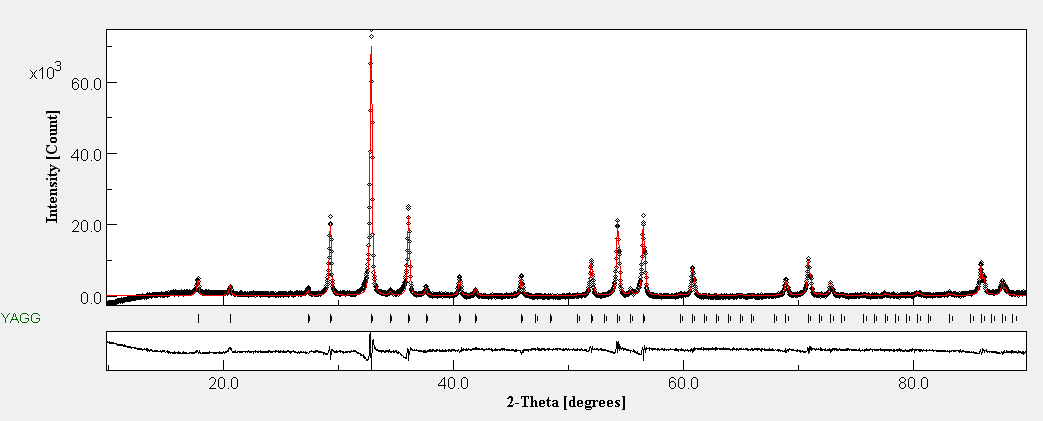


Figure S6. XRD patterns (dots) and the Rietveld fits (full lines) of the YAGG sample obtained at 1200° C for 1 h. Bar sequences of YAGG reference diffraction patterns and residual plot are shown along the bottom.


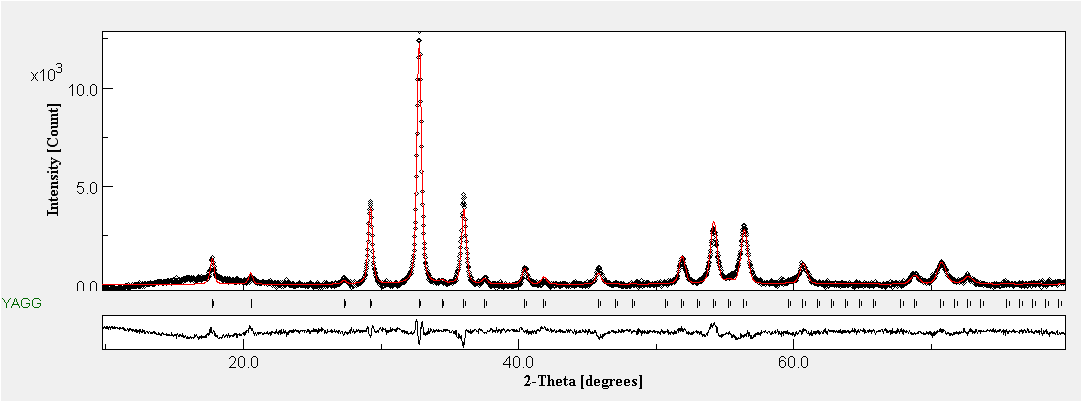


Figure S7. XRD patterns (dots) and the Rietveld fits (full lines) of the Y_3_(Al,Ga)_5_O_12_:Ce^3+^,Cr^3+^, Pr^3+^ sample obtained at 900° C for 1 h. Bar sequences of YAGG reference diffraction patterns and residual plot are shown along the bottom.


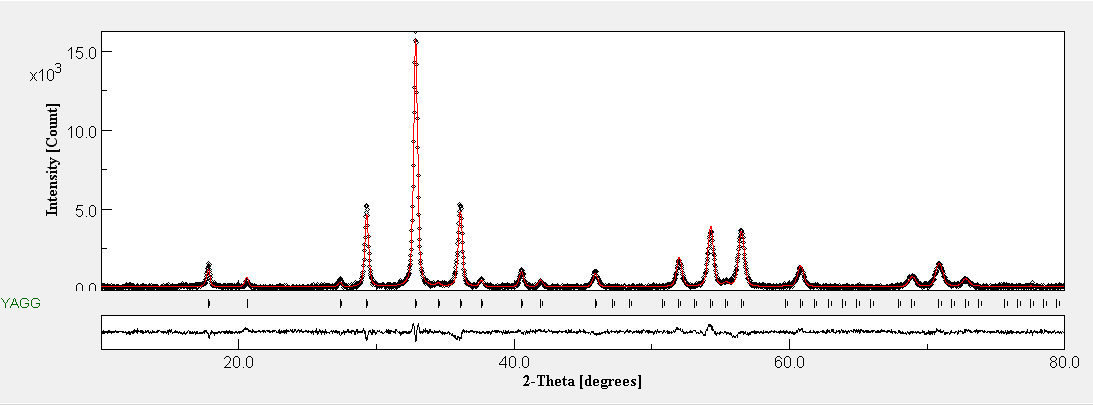


Figure S8. XRD patterns (dots) and the Rietveld fits (full lines) of the Y_3_(Al,Ga)_5_O_12_:Ce^3+^,Cr^3+^, Pr^3+^ sample obtained at 1000° C for 1 h. Bar sequences of YAGG reference diffraction patterns and residual plot are shown along the bottom.


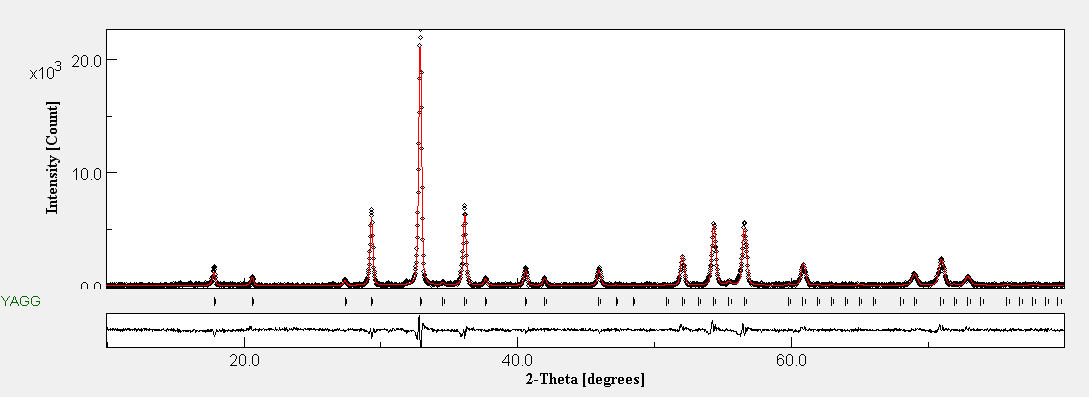


Figure S9. XRD patterns (dots) and the Rietveld fits (full lines) of the Y_3_(Al,Ga)_5_O_12_:Ce^3+^,Cr^3+^, Pr^3+^ sample obtained at 1100° C for 1 h. Bar sequences of YAGG reference diffraction patterns and residual plot are shown along the bottom.


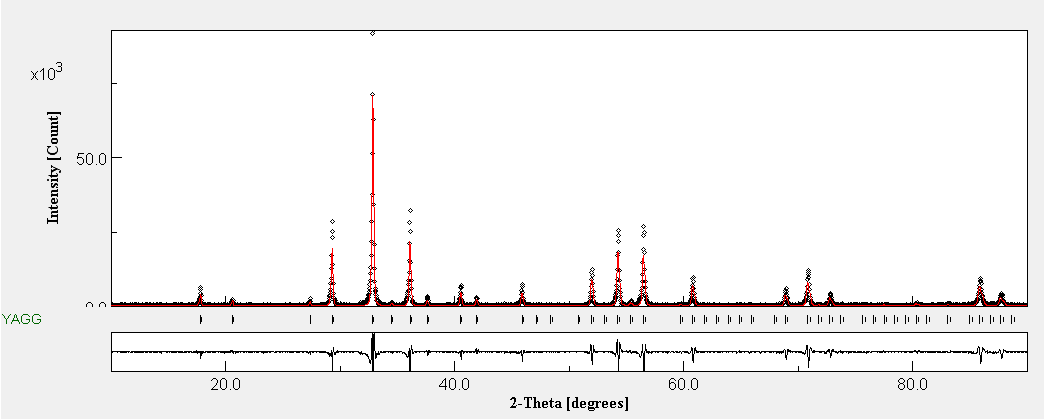


Figure S10. XRD patterns (dots) and the Rietveld fits (full lines) of the Y_3_(Al,Ga)_5_O_12_:Ce^3+^,Cr^3+^, Pr^3+^ sample obtained at 1200° C for 1 h. Bar sequences of YAGG reference diffraction patterns and residual plot are shown along the bottom.


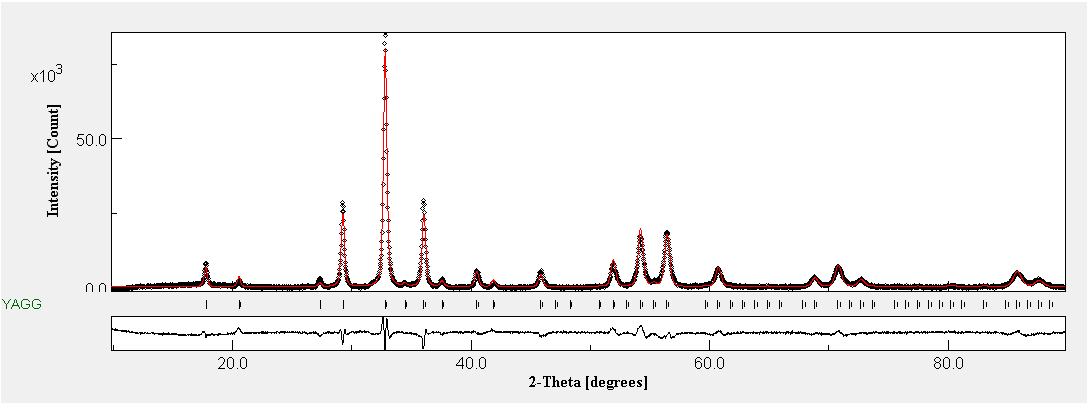


Figure S11. XRD patterns (dots) and the Rietveld fits (full lines) of the Y_3_(Al,Ga)_5_O_12_:Ce^3+^ sample obtained at 900° C for 1 h. Bar sequences of YAGG reference diffraction patterns and residual plot are shown along the bottom.


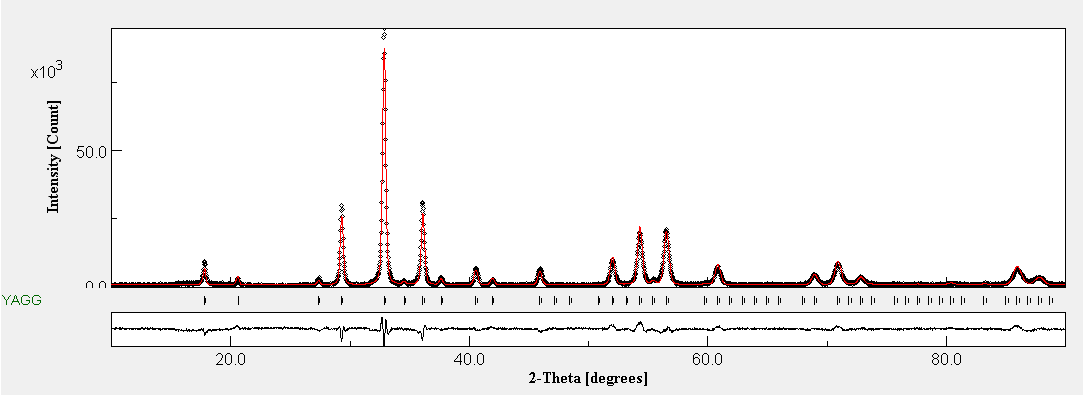


Figure S12. XRD patterns (dots) and the Rietveld fits (full lines) of the Y_3_(Al,Ga)_5_O_12_:Ce^3+^ sample obtained at 1000° C for 1 h. Bar sequences of YAGG reference diffraction patterns and residual plot are shown along the bottom.


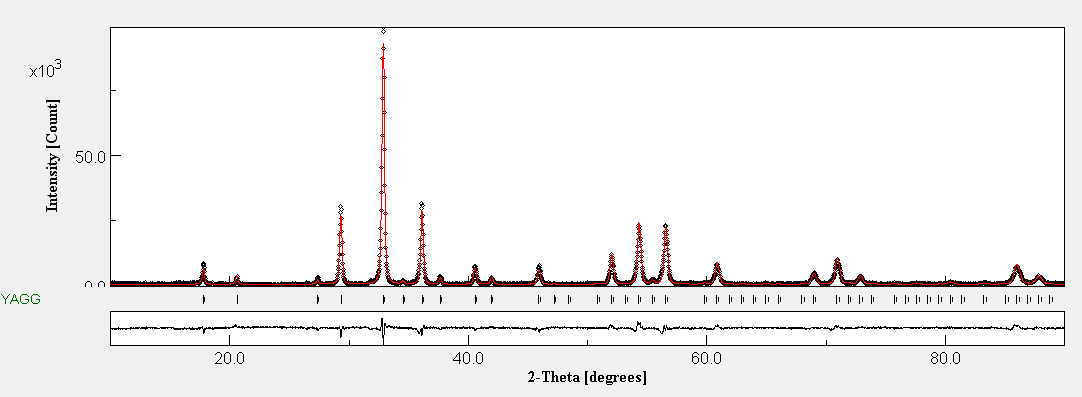


Figure S13. XRD patterns (dots) and the Rietveld fits (full lines) of the Y_3_(Al,Ga)_5_O_12_:Ce^3+^ sample obtained at 1100° C for 1 h. Bar sequences of YAGG reference diffraction patterns and residual plot are shown along the bottom.


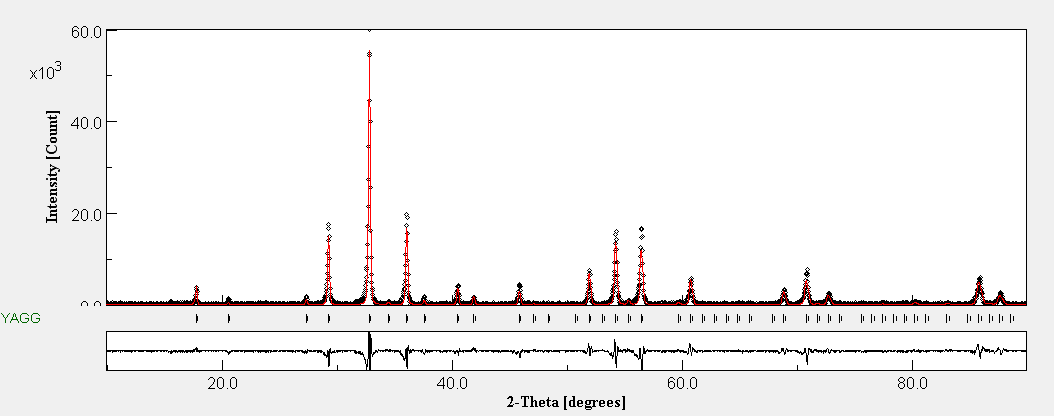


Figure S14. XRD patterns (dots) and the Rietveld fits (full lines) of the Y_3_(Al,Ga)_5_O_12_:Ce^3+^ sample obtained at 1200° C for 1 h. Bar sequences of YAGG reference diffraction patterns and residual plot are shown along the bottom.

| 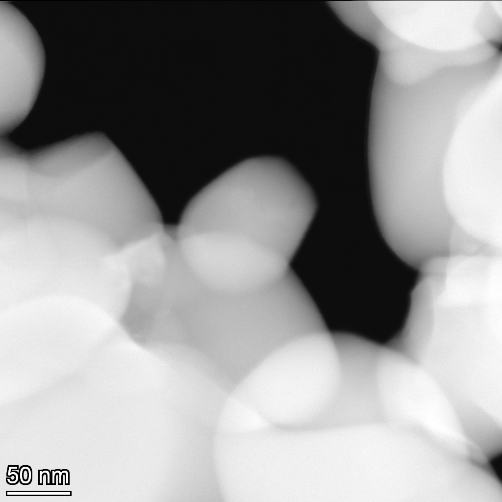 | **Raw EDS spectrum of selected area**  Element Atomic Fraction (%) Atomic Error (%)  O 49.86 5.28  Al 13.97 3.13  Ga 17.63 2.97  Y 18.54 3.08  **Elaborated data**  O 56.76 5.68  Al 11.19 2.48  Ga 15.13 2.50  Y 16.93 2.75 |
| --- | --- |

Figure S15. Elemental composition of the Y_3_(Al,Ga)_5_O_12_:Ce^3+^,Cr^3+^, Pr^3+^ sample obtained at 1100° C for 1 h obtained by EDS.





Figure S16. PLE spectra of YAGG:Ce^3+^ nanophosphors annealed at different temperatures.

λ_exc_: 520 nm at RT.

**

**

Figure S17. PLE spectra of YAGG:Ce^3+^,Cr^3+^,Pr^3+^ nanophosphors annealed at different temperatures. λ_exc_: 520 nm at RT.





Figure S18. PLE spectra of YAGG:Ce^3+^,Cr^3+^,Pr^3+^ nanophosphors annealed at different temperatures. λ_exc_: 606 nm at RT.

**

**

Figure S19. PLE spectra of YAGG:Ce^3+^,Cr^3+^,Pr^3+^ nanophosphors annealed at different temperatures. λ_exc_: 709 nm at RT.

**The luminescence decay curves and efficiency of energy transfer**

The emission decay curves of the YAGG:Ce^3+^ and YAGG:Ce^3+^,Cr^3+^,Pr^3+^ nanophosphors were measured for the emission of Ce^3+^ at 520 nm under the laser excitation at 450 nm (Fig. 1a and 1b respectively).

All the luminescence decay curves were analyzed and pre-prepared to cut off the laser line visible at the beginning of some curves. The subtraction had the same value for the same set of data and the spectra were not normalized for calculations. Then, the effective (average) decay times were calculated according to the formula:

$$\tau_{eff}=\frac{\int I\left( t \right)*t dt}{\int I\left( t \right) dt}$$

and the results are presented in function of annealing temperature (Fig.1c). This average decay time of the YAGG:Ce^3+^ nano-phosphors annealed at 900, 1000, 1100 and 1200° C are 83, 87, 69 and 57 ns respectively. While this decay time shortens to 81, 74, 52, and 50 ns when the phosphors are co-doped with Cr^3+^ and Pr^3+^. The shortening of the Ce^3+^ decay time is caused by the presence of additional paths to depopulate the Ce^3+^ 2D excited state in case of co-doped sample, *i.e.* the energy transfer to Cr^3+^ and/or Pr^3+^.

Energy transfer efficiency for the PL from Ce^3+^ to Cr^3+^ and Pr^3+^ in YAGG matrix can be calculated from the fluorescence lifetimes:

$$\eta=\left( 1-\frac{\tau_{DA}}{\tau_{D}} \right)*100\%$$

The obtained results as a function of annealing temperature are presented at Fig. 1c. The energy transfer efficiency from Ce^3+^ to Pr^3+^ and Cr^3+^ increases with annealing temperature increase up to 1100° C and consist 2 %, 15 % and 24 % respectively. With a further annealing temperature increase to 1200° C the energy transfer efficiency decrease and consist 11%.

**



**



Figure S20. The luminescence decay curves of (a) YAGG:Ce^3+^, (b) YAGG:Ce^3+^,Cr^3+^,Pr^3+^ and (c) average DT and efficiency of energy transfer. λ_exc_: 450 nm and λ_em_: 520 nm at RT.





Figure S21. PL and PersL CIE diagram of YAGG:Ce^3+^,Cr^3+^,Pr^3+^ phosphors annealed at 1100^o^ C

**

**

Figure S22. PersL decay curves of YAGG:Ce^3+^ nanophosphors annealed at different temperatures. λ_irr_: 450 nm (1 mWcm^-2^) for 5 min at RT and the observed signal intensity was corrected by mass.

**

**

Figure S23. PersL decay curves of YAGG:Ce^3+^,Cr^3+^,Pr^3+^ nanophosphors annealed at different temperatures. λ_irr_: 450 nm (1 mWcm^-2^) for 5 min at RT and the observed signal intensity was corrected by mass.















Figure S24. TL glow curve of (a) YAGG:Ce^3+^, (b) YAGG: Pr^3+^, (c) YAGG:Cr^3+^ and (d) YAGG:Ce^3+^,Cr^3+^,Pr^3+^ irradiated by X-ray (15 kV, 0.5 mA, 5 min) as well as (e) YAGG:Ce^3+^, (f) YAGG:Ce^3+^,Cr^3+^,Pr^3+^ irradiated by 450 nm (1 mWcm^-2^ 5 min) at RT.

Table S1. Shape factors (μ), activation energy (E) and frequency factor (s) for single doped and three doped powders after irradiation by X-Ray and blue-light sources.

| Sample | T (° C) | T_max_* (K) | μ | E (eV) | s (s^-1^) | Δs/s |
| --- | --- | --- | --- | --- | --- | --- |
| **X-ray irradiation** | | | | | | |
| YAGG:Ce^3+^ | 900 | 326 | 0.67±0,03 | 0.52±0.09 | 10^6^ | 3.6 |
|  | 1000 | 332 | 0.54±0,04 | 0.74±0.02 | 10^10^ | 0.5 |
|  | 1100 | 336 | 0.56±0,04 | 0.86±0.06 | 10^11^ | 2.3 |
|  | 1200 | 335 | 0.57±0,04 | 0.75±0.05 | 10^10^ | 1.8 |
| YAGG:Pr^3+^ | 900 | 341 | 0.72±0,02 | 0.70±0.08 | 10^9^ | 2.8 |
|  | 1000 | 338 | 0.61±0,03 | 0.69±0.04 | 10^9^ | 1.5 |
|  | 1100 | 335 | 0.65±0,03 | 0.67±0.05 | 10^9^ | 1.7 |
|  | 1200 | 333 | 0.60±0,03 | 0.68±0.06 | 10^9^ | 2.2 |
| YAGG:Cr^3+^ | 900 | 338 | 0.59±0,03 | 0.70±0.02 | 10^9^ | 0.7 |
|  | 1000 | 336 | 0.57±0,04 | 0.61±0.09 | 10^7^ | 3.4 |
|  | 1100 | 338 | 0.57±0,04 | 0.58±0.04 | 10^7^ | 1.6 |
|  | 1200 | 335 | 0.57±0,04 | 0.60±0.06 | 10^7^ | 2.1 |
| YAGG:Ce^3+,^Cr^3+^,Pr^3+^ | 900 | 332 | 0.59±0,03 | 0.64 ±0.05 | 10^8^ | 1.7 |
|  | 1000 | 331 | 0.55±0,04 | 0.71±0.03 | 10^9^ | 1.2 |
|  | 1100 | 334 | 0.55±0,04 | 0.74±0.01 | 10^10^ | 0.4 |
|  | 1200 | 329 | 0.53±0,04 | 0.73±0.02 | 10^10^ | 0.7 |
| **Blue light irradiation LD @ 450 nm** | | | | | | |
| YAGG:Ce^3+^ | 900 | 325 | 0.52±0,04 | 0.53±0.04 | 10^7^ | 1.7 |
|  | 1000 | 331 | 0.53±0,05 | 0.72±0.01 | 10^9^ | 0.4 |
|  | 1100 | 332 | 0.55±0,04 | 0.73±0.01 | 10^10^ | 0.4 |
|  | 1200 | 331 | 0.57±0,04 | 0.79±0.03 | 10^11^ | 1.1 |
| YAGG:Ce^3+,^Cr^3+^,Pr^3+^ | 900 | 327 | 0.55±0,04 | 0.70±0.01 | 10^9^ | 0.4 |
|  | 1000 | 334 | 0.53±0,04 | 0.59±0.09 | 10^7^ | 3.3 |
|  | 1100 | 330 | 0.55±0,04 | 0.73±0.03 | 10^10^ | 1.1 |
|  | 1200 | 329 | 0.51±0,05 | 0.71±0.04 | 10^9^ | 1.7 |
| ** T_max_ - value of temperature at maximum TL intensity for β = 1 Cs^-1^* | | | | | | |
